# Supplementary material for: Quantifying local stability and noise levels from time series in the US Western Interconnection blackout on 10th August 1996
Source: Nat Commun. 2025 Jul 7;16:6246. doi: 10.1038/s41467-025-60877-0 (PMC12234730; doi:10.1038/s41467-025-60877-0)
Supplement: Supplementary file 1 — Supplementary Information [file 41467_2025_60877_MOESM1_ESM.pdf]

Supplementary Information for

Quantifying local stability and noise levels from time series in the US Western Interconnection blackout on 10th August 1996

Martin Heßler[1,2,*] **and Oliver Kamps**[1]

[1]Center for Nonlinear Science, University of Münster, Corrensstraße 2, 48149 Münster, Germany
[2]University of Münster, Wilhelm-Klemm-Straße 9, 48149 Münster, Germany
[*]m_hess23@uni-muenster.de

Corresponding author: Martin Heßler
E-mail: m_hess23@uni-muenster.de

This pdf includes:

- Supplementary text S1 to S19
- Supplementary Boxes S1 to S3
- Figures S1 to S15
- Tables S1 to S5

Other supplementary materials for this manuscript include the following:

- Data and simulation codes are provided in the GitHub repository[1] at https://github.com/MartinHessler/Disentangling_Tipping_Types.
- The open-source package *antiCPy*[2,3] is available at https://github.com/MartinHessler/antiCPy under a *GNU General Public License v3.0* and documented at https://anticpy.readthedocs.io.

Supplementary Information: Text and Figures

S1 Limits of Non-Parametric Leading Indicators

In Figure S1, two example scenarios of critical transitions with and without changing noise level are analysed. The time series y in the upper graphs of Figures S1(a,b) and (c,d) originate from models, exhibiting a pitchfork and a fold bifurcation, respectively, as in Equations 2 and 3. Simulation details are outlined in SI S2.

The four time series have in common that they exhibit destabilizing control parameter shifts that finally lead to B-tipping. The two time series in Figure S1(a,c) undergo a pure B-tipping event due to a linear control parameter shift, whereas the counterparts in Figure S1(b,d) are additionally influenced by (b) decreasing and (d) increasing noise levels. However, these distinctly more complex and more realistic scenarios generate rather similar time series. For example, the time series in Figures S1(c,d) look very similar. Also the second half of the time series in S1(b) is comparable to the time series in Figure S1(a). To accommodate the reading flow the model equations and a detailed description of the simulation procedure can be found in Section S2.

Only under rather restrictive conditions, the non-parametric leading indicators are able to announce an emerging transition. For example, the parameter shift must be slow and smooth to be followed by a statistical rolling window approach, the sampling rate needs to be high, the noise levels should be small, the choice of the observable is crucial and much more.[4–8] But more important is the fact that statistical measures as AR1 $\hat{\rho}_1$ and STD $\tilde{\sigma}$ do not provide information about the origin of their raise, do not distinguish between pure B-tipping and more complex destabilization scenarios which, e.g., involve a variation of the internal noise. Thus, they can easily be ambiguous: If we stay with the non-parametric reasoning, the standard leading indicators AR1 $\hat{\rho}_1$ and STD $\tilde{\sigma}$ should increase *both* at the same time because of CSD to be regarded as significant signs of B-tipping (cf. also Section S9), as visible in Figure S1(a). However, the same system driven by decreasing noise level (approximately the red dotted line in the lower graph of Figure S1(b)) still destabilizes by B-tipping, but with an ambiguous convex shape of the STD $\tilde{\sigma}$.

The qualitative indicators computed from the time series in Figures S1(a,c,d) result in a similar signature, although the data generating processes of the time series in (a,c) and (d) are different. The time series in (d) is driven by increasing endogenous disturbances, in contrast to the time series in Figures S1(a,c), which are purely destabilizing due to B-tipping. The AR1 $\hat{\rho}_1$ and STD $\tilde{\sigma}$ do not provide information about the decreasing or increasing noise levels of the systems in Figures S1(b,d).

Note that it can have severe consequences if an increasing noise level during an approaching B-tipping event is not detected because it may cause a potentially higher N-tipping probability in multistable systems which can result into a transition significantly earlier than the time at which the actual B-tipping point would be reached. In this sense, the overall noise level has to be small enough to avoid N-tipping far before a B-tipping point if control and management decisions should be justified on AR1 $\hat{\rho}_1$ and STD $\tilde{\sigma}$.

In contrast to these findings for the AR1 $\hat{\rho}_1$ and STD $\hat{\sigma}$, the lower plots in our examples of Figure S1 reveal the potential of the BLE to detect the interplay of drift destabilization and changing noise level in the observed data at the same time. In the cases (a,c) the noise level (shown in red with green CB) stays constant, but the slope as leading indicator (in blue with orange CBs) approaches zero, which indicates the ongoing B-tipping destabilization correctly. Whereas the AR1 $\hat{\rho}_1$ and the STD $\tilde{\sigma}$ can only detect the B-tipping transition in Figure S1(d), the approaching B-tipping event in Figures S1(b) and (d) is correctly reflected by the increasing drift slope ζ. Furthermore, in contrast to the AR1 $\hat{\rho}_1$ and the STD $\tilde{\sigma}$, the decreasing/increasing noise level (marked by the red dotted lines), i.e., the changing N-tipping probability in multistable systems, is captured by the noise level estimates $\hat{\sigma}$ of the BLE in the lower graphs of Figures S1(b,d), respectively.

S2 Simulation Details of the Introductory Examples

For the sake of readability, the simulation details of the pitchfork and fold model, shown and analysed in Figures S1 and S6, are summarized in the following. The pitchfork models in Figure S1(a,b) follow the model Equations 2 and undergo a static bifurcation from a one-peaked to a double-peaked PDF[9]. In the purely B-tipping case (cf. Figure S1(a)), the control parameter is linearly shifted from 10 to -2, with $3 \cdot 10^4$ data samples simulated over the time interval $[0, 2000]$, after discarding the initial 500 samples as a burn-in period. The noise level is chosen constant as $\sigma = 0.15$. In order to create the similar looking dataset with decreasing noise level, shown in Figure S1(b), the control parameter of the pitchfork model is linearly shifted from -27.5 to 6.05 during the simulations. Furthermore, the noise level is adapted corresponding to the distribution[10]

$$p(x) \propto \exp\left(\frac{-2V}{Q}\right) \tag{S.1}$$

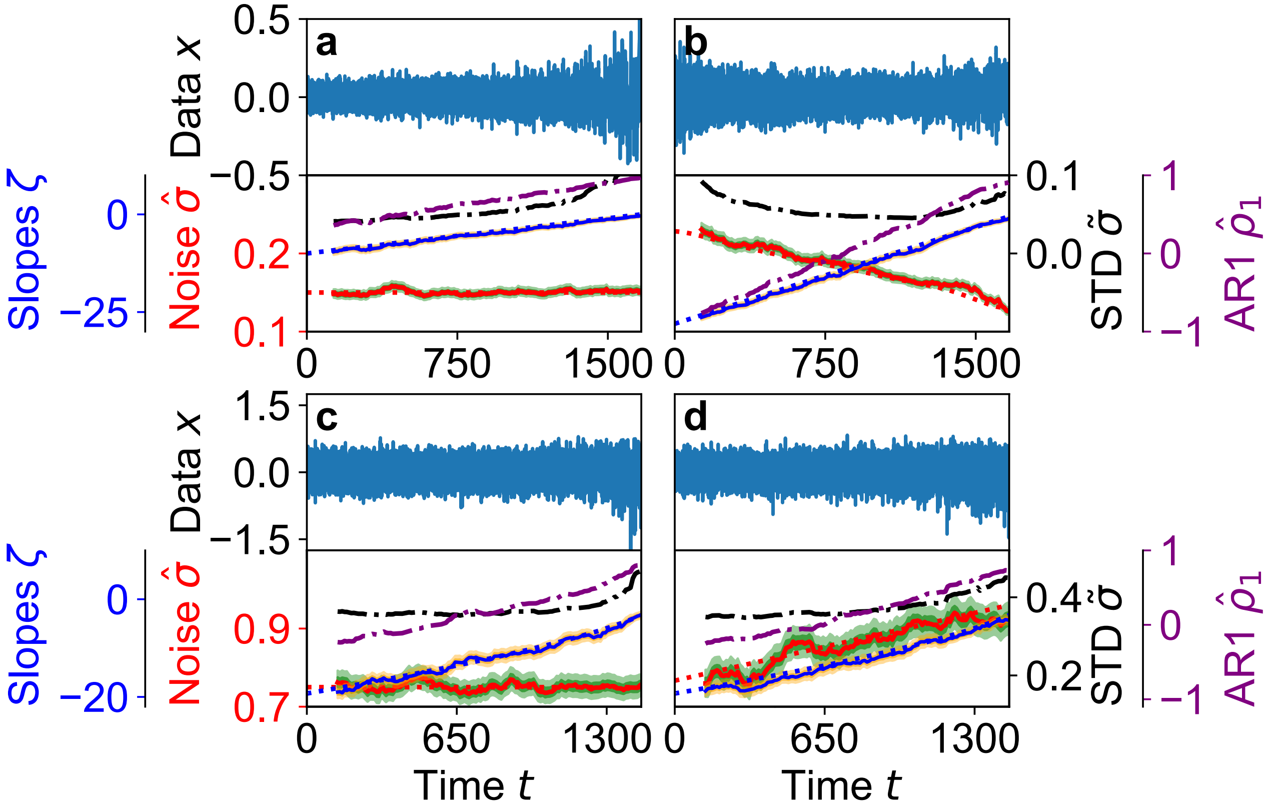

Figure S1. **(a,b)** Two examples of time series pairs that are similar in the second half, and **(c,d)** pairs that are similar over the entire range. The time series in **(a,c)** originate from pure B-tipping destabilization. In contrast, the data in **(b,d)** stem from combined B-tipping with decreasing or increasing noise levels, respectively. In example **(a,b)**, we refer to the static bifurcation definition of Crauel et al[9]. The leading indicators are computed in rolling windows and shown in the lower graphs. **(a,c)** The drift slope estimates $\hat{\zeta}$ (blue with orange CIs) approach zero, while the noise level estimates $\hat{\sigma}$ (red with green CIs) are constant. The estimates match the corresponding ground truths (blue and red dotted lines for drift slopes and noise levels, respectively). **(b,d)** The decreasing and increasing noise counterparts are well captured by the BLE results. In contrast, the non-parametric AR1 $\hat{\rho}_1$ and STD $\tilde{\sigma}$ exhibit nearly identical fingerprints, specifically positive trends, across three different destabilization scenarios **(a,c,d)**, and thus do not provide insights into the ongoing dynamical processes. Furthermore, they are not applicable in example **(b)** because a convex STD $\tilde{\sigma}$ curve cannot be unambiguously interpreted as a leading indicator. In principle, the qualitative indicators can be transformed into linear parametric estimates of the BLE by assuming an *Ornstein-Uhlenbeck* (OU) process to explain the data. However, in Section S9, this first approximation fails over large time intervals when AR1 $\rho_1 < 0$, during which the estimates diverge to infinity. Additionally, the estimates are strongly biased.

of the related Fokker-Planck equation[10] of the time series in Figure S1(a), with the potential

$$V(x) = -\int_{x_0}^{x} h(x)\mathrm{d}x \tag{S.2}$$

and the diffusion coefficient

$$Q = \frac{1}{2}g^2(x), \tag{S.3}$$

where $h(x)$ and $g(x)$ are the drift and diffusion, respectively, of the Langevin Equation 1. The very noisy results of this noise level estimates $g(x)$ and a version that is smoothed via a Gaussian kernel with a kernel width of $\sigma_\mathrm{k} = 2{\cdot}10^3$ points (i.e., $\sigma_\mathrm{k} = 133.3\,\mathrm{a.u.}$) are shown as blue and red lines, respectively, in Figure S2. The smoothed version is also shown as a red dotted line in Figure S1(b) to guide the eye. The pitchfork models are analysed until $\nu \geq 0$.

The fold model is computed via the model Equations 3. The purely B-tipping version of Figure S1(c) is simulated over the time range $[0, 2000]$ with $3{\cdot}10^4$ data samples, with a linear shift of the control parameter r from 15 to -2 and the noise level

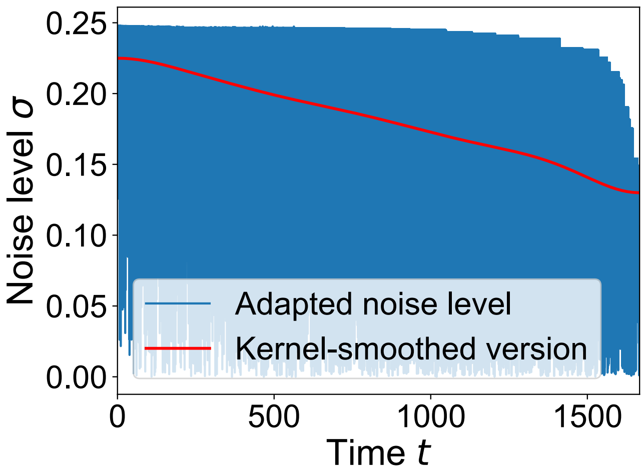

Figure S2. The computed noise level $g(x)$ for the pitchfork model is shown. The control parameter ν is linearly increased over the range $[-27.5, 6.05]$ and the noise $g(x)$ is adjusted to match the data distribution of Equation S.2 for the time series in Figure S1(a). A version smoothed via a Gaussian kernel with kernel width $\sigma_k = 2\cdot 10^3$ points (i.e., $\sigma_k = 133.3\,\text{a.u.}$) is shown in red. It is also shown in Figure S1(b) as red dotted line.

$\sigma = 0.75$. The time series in Figure S1(d) is simulated similarly, but with a linearly increasing noise level over the range $[0.7, 0.9]$. The fold models are analysed until $r \leq -0.5$. The cut versions are detrended by subtracting a Gaussian kernel smoothing with kernel width $\sigma_k = 1.5\cdot 10^3$ points (i.e., $\sigma_k = 100\,\text{a.u.}$).

S3 Relationships of Power Grid Models With Different Levels of Coarse-Graining to Understand the Western Interconnection Cascading Failure

In stable operation, the frequency across the entire grid is equal to the center of inertia or bulk frequency $\bar{\omega}$, which is described by the *aggregated swing equation* (ASE)[11–14]

$$\dot{\bar{\omega}} = -\bar{\gamma}\cdot\bar{\omega} + \bar{\sigma}\bar{\xi}(t). \tag{S.4}$$

To avoid confusion, please note the small differences in the notation and scaling compared to Schaefer et al.[12]. The authors denote the angular velocity by ω with the frequency f. In the following, the frequency is denoted directly by ω, and the 2π scaling from the original equation is omitted. Essentially, this *macroscopic* model of the frequency dynamics represents an OU process with a macroscopic restoring rate $\bar{\gamma}$, noise amplitude $\bar{\sigma}$, and an arbitrary stochastic process $\bar{\xi}$ that encapsulates fast dynamics across all non-resolved scales. Comparing the (N)BLE parameterisations, the drift slopes $\hat{\zeta}_{(\text{NBLE})}$ and noise amplitudes $\hat{\sigma}$ ($\hat{\Psi}$) are therefore closely related to the ASE's restoring rate $\bar{\gamma}$ and noise amplitude $\bar{\sigma}$, respectively. Note that Schaefer et al.[12] point out that $\bar{\gamma}$ includes lower scale contributions of primary control actions, but also from damper windings and related equipment. This is in line with the observations from the analysed real-world NAWI outage (cf. Results). However, the relationship between the macroscopic frequency dynamics and lower scale descriptions of the high-dimensional power grid state $\underline{x}$, hypothetically extending down to individual grid participants (cf. Supplementary Box S1 for some examples) is complex and not straightforward. Thus, in the following, additional results are provided to discuss possible effects on the macroscopic ASE description from lower descriptive scales.

Up to now, a microscopic description of nonlinear interactions between individual grid participants and their fundamental principles is out of reach for numerical and technical limitations. This represents an issue of overwhelming complexity. In addition, at a hypothetical first-principle descriptive level—i.e., where the dynamics and interactions of each individual component are described by fundamental physical laws—the formalism of the Langevin equation does not apply. A Langevin equation reduces the system dynamics to key effects on a slow scale and summarizes all faster degrees of freedom as stochastic contributions. This hypothetical first-principle description might not be stochastic at all, if the involved fundamental laws do not encompass real natural randomness.

Nevertheless, a lifting from the unfeasible microscopic model perspective to a mesoscopic level is possible: Therein, generators and consumers or combined bunches of them are considered as nodes in a power network. These nodes can be modelled by nonlinear Kuramoto oscillators[15]. Including damping and noise as well as the dynamics of the phase Φ_i for N nodes, leads to a

system of second-order *stochastic differential equations* (SDEs) given by

$$\dot{\phi}_i = \omega_i \tag{S.5}$$

$$M_i \dot{\omega}_i = P_i + \sigma_i \xi_i - D_i \omega_i + \sum_{j=1}^{N} K_{ij} \sin(\phi_j - \phi_i). \tag{S.6}$$

The nodes' frequencies ω_i are defined as $\omega_i = \omega_i^{(\mathrm{abs})} - \omega_{\mathrm{R}}$ with the absolute frequency $\omega_i^{(\mathrm{abs})}$ and the reference frequency ω_{R} ($\omega_{\mathrm{R}} = 60\,\mathrm{Hz}$ for the NAWI). Every node i features the inertia M_i, the phase angle ϕ_i, mechanical power P_i, random noise ξ_i with noise level σ_i, damping D_i, and the coupling matrix entries K_{ij}, which reflect topological features of the grid.[12] The SDE system is called *(classical) swing equation* (CSE) or *second order Kuramoto(-like) model*.[12,16] These models are mainly used to investigate topological grid features and the grid's ability of self-synchronization (for a prominent example, cf. Ref.[16]).
For mainly two reasons, a model of this type can be helpful to fill the gap between individual components and macroscopic frequency dynamics as well as between different levels of coarse-grained descriptions to some extent:

1. As it is located at a descriptional mesoscale, it is closer related to the microscopic level of individual components.

2. The macroscopic ASE S.4 can be derived from the mesoscopic CSE S.5.[12] Therefore, a homogeneous damping-inertia ratio $\bar{\gamma} = \frac{D_i}{M_i}$, symmetric coupling $K_{ij} = K_{ji}$, and power balance $\sum_{i=1}^{N} P_i \equiv 0$ are assumed. This leads to the ASE with the bulk frequency $\bar{\omega} = \sum_{i=1}^{N} M_i \omega_i / \sum_{i=1}^{N} M_i$. In particular, $\bar{\gamma} = \frac{D_i}{M_i}$ clarifies that the macroscopic frequency restoring rate integrates damping and primary control actions from mesoscale levels. In contrast to the second order Kuramoto model, the ASE does not explicitly model damping because it is a first order SDE, but captures it instead by $\bar{\gamma}$.

In Figure S3, a preliminary mesocale model reconstruction of three key episodes of the NAWI outage is considered to provide further evidence for the real-world findings and to improve the understanding of the mesocale-macroscale relationships. Furthermore, first systematic calculations for isolated parameter changes in the CSE are conducted. The CSE S.5 with $i \in G, \bar{G}, \bar{C}$ is used to realize the scenarios (i) to (iii) in Figure S3(a), i.e.:

(i) fast-scale disturbances due to the pre-outage conditions,

(ii) the 500 kV Keeler-Allston line tripping due to a THIF,

(iii) and the loss of the McNary power units with subsequent failure of the damping equipment.

The modelling attempts are oriented on statements from the approved Western Systems Coordinating Council Disturbance Report[17], aiming to identify the simplest models that align with the report's information and capture the qualitative characteristics of the observed (N)BLE fingerprints. Since Scenarios (ii,iii) involve tripping of a line or node, it is necessary to consider at least a three-node system with two lines. In this framework, the symbols $\bar{C}$ and $\bar{G}$ represent the aggregated consumers and generators of the global NAWI grid. In Scenario (ii), the line to the generating node G is interpreted as the 500 kV Keeler-Allston line, while in Scenario (iii), the generator G corresponds to the McNary power units. This approach results in a simplified model, which largely ignores the topological features of the NAWI. Nevertheless, it is sufficient to qualitatively reconstruct the real-world fingerprints that are observed (cf. Results) and to provide a foundation for further discussion.
Since a strongly simplified model is considered, the parameter choices cannot be determined through realistic approximations. Therefore, the simulated data are not expected to match the orders of magnitude given by the time series $\tilde{\omega}_{\mathrm{P}}(t)$. Instead, the focus is on the numerically important facts, such as ensuring a synthetic frequency time sampling step $\Delta t = 5 \cdot 10^{-2}$a.u. in all scenarios, which matches the time step of the original pre-outage time series ($\Delta t = 5 \cdot 10^{-2}$s). Additionally, the periodicity of 0.2 Hz to 0.4 Hz (cf. SI S15) present in the original pre-outage time series roughly coincides with the periodicities in the data from Scenarios (i–iii), with frequencies ranging from $\sim 0.2\,\mathrm{Hz}$ to 0.8 Hz. We refer to the insets in Figures S3(b,f,j) as examples of these oscillations.
Every model time series undergoes the same preprocessing as the real-world time series (cf. Results): A slow trend is fitted using a Gaussian kernel with a bandwidth $\sigma_{\mathrm{k}} = 5\,\mathrm{s}$ and subtracted from the raw data to obtain the detrended model frequency time series $\tilde{\omega}_i$. The (N)BLE priors and the window shift of 100 points are set exactly as in the real NAWI frequency analyses. The only change is in the window size, which is chosen as $N_{\mathrm{w}} = 8 \cdot 10^3$ to stabilize the (N)BLE against the significantly higher model fluctuations. However, the model results are robust to notable window size variations, as shown in SI S16.
The simulation details, (N)BLE parameter, and the parameter choices of Scenarios (i–iii) are summarized in Tables S1 and S2. All simulations start under stable grid conditions that fulfil the assumptions of the ASE, and the signal $\omega_{\bar{G}}$ is analysed in all scenarios.

Scenario	Burn-In Samples	Time Interval	Total Samples	Thinning N_{thin}	Signal of Node
**(i)**	$1.5 \cdot 10^5$	$[0, 2000]$	$4 \cdot 10^6$	100	G
**(ii)**	$8.0 \cdot 10^4$	$[0, 2000]$	$4 \cdot 10^6$	100	$\bar{G}$
**(iii)**	$8.0 \cdot 10^4$	$[0, 4000]$	$8 \cdot 10^6$	100	$\bar{G}$

Table S1. Simulation details for the NAWI key outage scenarios shown in Figure S3. A burn-in phase with constant initial parameters is excluded before $t = 0$ in every simulation to account for transient effects. The thinning N_{thin} indicates that every N_{thin}-th sample is selected to create the measured frequency signals.

Scenario	Parameter	Node Index		
		Consumers $\bar{C}$	Generators $\bar{G}$	Generator G
**(i)**	M_i	1.5	1.5	1.5
	P_i	-6	3	3
	D_i	0.25	0.25	0.25
	$K_{\bar{C}i}$	—	8	8
	σ_i	$[0.1, 0.6]$	0.1	0.1
	$\xi(t)$	$\Gamma(t)$	$\Gamma(t)$	$\Gamma(t)$
**(ii)**	M_i	1	1	1
	P_i	-5	$P_{\bar{G}} = \begin{cases} 2.5 & \text{for } t \in [0, 1400) \\ 5 & \text{for } t \in [1400, 2000] \end{cases}$	$P_G = \begin{cases} 2.5 & \text{for } t \in [0, 1400) \\ 0 & \text{for } t \in [1400, 2000] \end{cases}$
	D_i	0.25	0.25	0.25
	$K_{\bar{C}i}$	—	$K_{\bar{C}\bar{G}} = \begin{cases} 7 & \text{for } t \in [0, 500] \\ [7, 50] & \text{for } t \in (500, 1000] \\ 50 & \text{for } t \in (1000, 2000] \end{cases}$	$K_{\bar{C}G} = \begin{cases} 7 & \text{for } t \in [0, 1400) \\ 0 & \text{for } t \in [1400, 2000] \end{cases}$
	σ_i	0	0.015	0
	$\xi(t)$	—	$\Gamma_{\text{red}}(t)$	—
**(iii)**	M_i	1	1	1
	P_i	-20	$P_{\bar{G}} = \begin{cases} 10 & \text{for } t \in [0, 2500] \\ [10, 20] & \text{for } t \in (2500, 2550] \\ 20 & \text{for } t \in (2550, 4000] \end{cases}$	$P_G = \begin{cases} 10 & \text{for } t \in [0, 2000] \\ 0 & \text{for } t \in (2000, 4000] \end{cases}$
	D_i	0.5	0.5	0.5
	$K_{\bar{C}i}$	—	25	$K_{\bar{C}G} = \begin{cases} 25 & \text{for } t \in [0, 2000.005) \\ 0 & \text{for } t \in [2000.005, 4000] \end{cases}$
	σ_i	0.25	0.25	0
	$\xi(t)$	$\Gamma(t)$	$\Gamma(t)$	—

Table S2. CSE parameters for modelling the key Scenarios (i–iii) of the NAWI outage, analysed in Figure S3. The parameter intervals denote linear tuning. Key parameters that are tuned over time are also drawn in Figure S3, and it holds $K_{ij} = K_{ji}$. The red noise $\Gamma_{\text{red}}(t)$ is generated using the OU process $\dot{\Gamma}_{\text{red}} = \dot{y} = -\frac{1}{0.25^2} \cdot y + 0.25\Gamma(t)$. The simulation details and parameters for the (N)BLE are provided in Table S1.

S3.1 Increased Pre-Outage Noise Levels

We begin our discussion with the pre-outage conditions characterized by increased fast-scale disturbances. The noise levels during the late restoration interval are roughly one order of magnitude lower than those during the early pre-outage period, already indicating a significantly increased system stress with dominant fast-scale effects on the frequency. While the exact origin of the higher noise level can only be hypothesized, the approved report[17] (cf. also Figure 1) suggests that a combination of factors may have played a role. Specifically, the report mentions a significant increase in electricity demand due to a heat wave, which led to intensive usage of air conditioning, as well as active energy trading driven by favourable conditions for hydroelectric power generation. These factors may have contributed to an amplification of the fast-scale dynamics, modelled here as an increasing absolute mesocale noise level $\sigma_{\bar{C}}$ affecting the consumer section node $\bar{C}$.

The time series and (N)BLE results are shown in Figures S3(b–d). The amplified mesoscale noise levels are integrated into the macroscopic frequency noise level estimates of the (N)BLE. It is emphasized that these estimates are expected to differ quantitatively from the mesoscale noise $\sigma_{\bar{C}}$, since using the (N)BLE essentially provides an estimate of the macroscopic ASE's noise level rather than the mesoscale noise level. The macroscopic (N)BLE approach resolves this *absolute* noise increase at the CSE's mesoscale. As expected from the Langevin form, the fast-scale dynamics do not affect the drift slopes $\hat{\zeta}_{(\mathrm{NBLE})}$. This serves as a prototypical case for the simplified conceptual reasoning of N-tipping illustrated in Supplementary Box S1(d). In this context, the power grid state $\underline{x}$ is closely related to the one-dimensional dynamics of the frequency $\omega(t)$.
Note that in the specific case of the NAWI blackout in 1996, the noise increase is likely attributable to the consumer section. However, repeating the analysis with noise fed into the generator section (cf. Figure S3)(a) yields almost identical results. This alternative scenario may represent the increasingly significant issue of low-inertia stochastic dynamics associated with renewable energy sources[13]. In this context, the asymmetry of the noise feed did not notably change the qualitative results.

S3.2 The Keeler-Allston Line Tree-Related High-Impedance Fault

Although the CSE lives on a lower descriptive scale than the ASE, modelling a *tree-related high-impedance fault* (THIF) on this scale is not straightforward. The detection and modelling of THIFs are complex research topics on their own[18, 19]. In essence, THIFs disturb the functionalities of the affected lines, which connect individual nodes of the power grid. Therefore, the focus is on a capacity change within the model. From the onset of a THIF until it is fully established, it can take a few seconds to minutes, during which the ongoing processes are complex. Initially, sporadic contact or high resistance from dry wood may result in a lower energy transmission through the affected line. As time progresses, a permanent contact is established, and subsequent carbonisation significantly decreases the tree's resistance. Ultimately, this leads to the formation of a near short circuit, severely impacting the energy transmission capacity of the line and resulting in extremely high currents. These currents can damage equipment if the THIF is not timely handled by the system protection protocol, which typically isolates and trips the affected line. However, until the line is isolated, its transmission capacity decreases continuously relative to the surrounding lines. Simultaneously, these surrounding lines must compensate for the THIF and experience typically higher systemic stress. Against this background, both grid lines are initially defined with equal capacities and the progressive degradation of the 500 kV Keeler-Allston line's functionality is modelled by raising the non-affected compensating line's transmission capacity (cf. Figure S3(e)). In doing so, the Keeler-Allston line's coupling weakens relative to that of the compensating grid line, which becomes more important for the grid's stability and synchronization.
Interestingly, the distinct drift slope trends observed between the BLE and NBLE in the real-world data (cf. Figure S13) are not reproduced when white noise $\Gamma(t)$ is added to the system. When white noise is used, both the BLE and NBLE exhibit the same anti-correlated trend in the (N)BLE estimates, showing increased local stability (i.e., more negative drift slopes) and noise level, similar to the BLE results in Figure S3(f).
Instead, a red noise signal $\Gamma_{\mathrm{red}}(t)$, with a correlation length oriented on the estimated real-world parameters of the NBLE in Table S2, is included via the aggregated generators $\bar{G}$ (cf. Table S2). Adding red noise reconstructs the anti-correlated behaviour of the BLE results, whereas the NBLE estimates decouple, as observed in the real-world frequency time series. Note that the mesoscopic noise amplitudes σ_i are fixed in this example, yet we observe an increase of the macroscopic (N)BLE noise levels $\hat{\sigma}$ and $\hat{\Psi}$. In this context, the noise level estimates highlight the greater impact of the mesoscopic fast-scale processes on the macroscopic ASE scale of the frequency dynamics. Importantly, these changes are not due to an absolute increase in the mesoscopic noise level, as seen in Scenario (i).
This result can be interpreted as follows: The capacities K_{ij} represent the formal coupling strengths between the nodes. As the capacity of the compensating grid increases, fast-scale disturbances propagate more rapidly through the grid. Simultaneously, the nodes react more strongly to incoming signals, including disturbances, due to the enhanced coupling. In consequence, the manifest macroscopic frequency noise level rises. This situation can increase the chance for N-tipping of the frequency, stemming from the amplified impact of previously suppressed mesoscopic noise contributions. Therefore, this observation is denoted as a *relative* macroscopic ASE noise increase to distinguish it from Scenario (i). Nonetheless, further research is

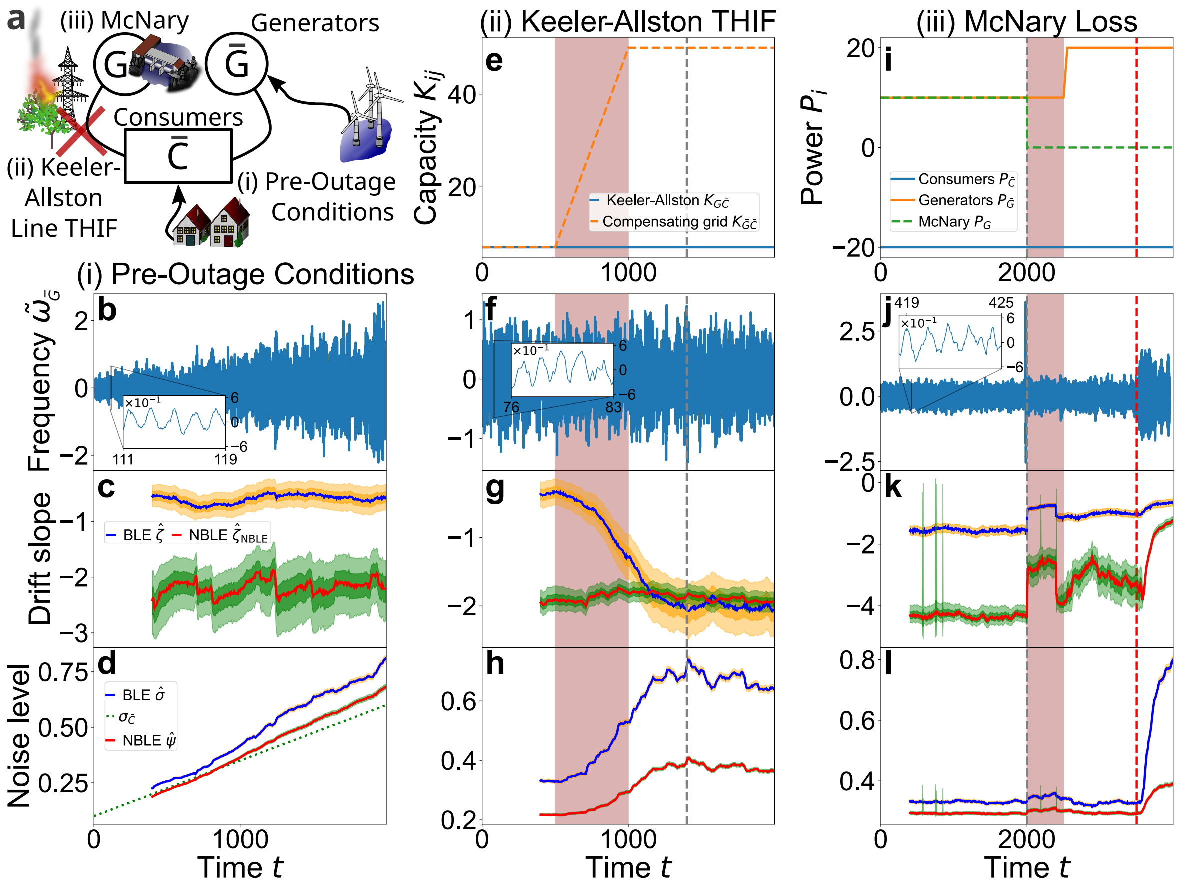

Figure S3. The analyses of the signal $\omega_{\bar{G}}$ from the simple CSE S.5 model scenarios in **(a)** qualitatively reproduce key characteristics in the (N)BLE trends during the NAWI cascading failure. Whereas the data amplitudes are not matched with the real-world magnitude due to the simplicity of the conceptual CSE model, the real-world periodicity of 0.2 Hz to 0.4 Hz is roughly captured by the simulated data with periodicities ranging from ~ 0.2 Hz to 0.8 Hz. The insets in Figures S3(b,f,j) illustrate the periodic characteristics present in the simulations. Simulation and (N)BLE details are summarized in Tables S1 and S2. **(b–d)** Scenario (i) represents the pre-outage conditions characterized by increased fast-scale influence in the NAWI. The ASE noise level estimates $\hat{\sigma}$ and $\hat{\Psi}$ quantify the absolute increase of the CSE noise level $\sigma_{\bar{C}}$, which also applies to tuning the noise levels of the generator nodes rather than those of the consumer section. **(e–h)** The disturbed transmission capabilities of the Keeler-Allston line are represented by a capacity increase of the compensating system. A simulation with red noise instead of white noise reproduces the observed behaviour of the BLE and NBLE drift slopes during the event (cf. Figure S13). The ASE noise levels in **(h)** increase, whereas the CSE noise amplitudes remain fixed. The relative noise increase on the macroscale means a potentially higher chance for N-tipping in the frequency dynamics. Stronger coupling on the mesoscale appears to cause faster noise propagation and impact in individual nodes. The grey dashed line marks the line tripping, which has no significant influence in this scenario. **(i–l)** The loss of the McNary units causes a power imbalance in the system. Also after the sharp step of one window length, the drift slope stays increased in windows not disturbed by the spikes of the McNary loss or by the subsequent power re-balancing, indicating reduced frequency stability in the meta-stable frequency regime. A primary control action is simulated to re-stabilize the frequency and to isolate the influence of failing damping equipment, marked by the red dashed line. The (N)BLE drift slopes and noise estimates rise sharply because deterministic and stochastic components of the signal are less damped. The degradation of stability and the relative noise increase of the ASE finally led to the islanding events during the NAWI cascading failure. Note that the less damped period is simulated in a subcritical regime to show the qualitative influence on the (N)BLE ASE estimates.

necessary to understand how general this behaviour is with respect to nonlinearities present in the system. Note that the actual tripping of the line had no significant influence on the simulations. Initially, it was placed at the end of the red interval, since that better fits the actual time of the Keeler-Allston line tripping. However, in the shown example, it is preferred to place it more than a window length apart from the capacity shift to demonstrate that there is no clear and direct (N)BLE response (cf. grey dashed line). Regardless of the line tripping position, the results were almost the same. The small BLE drift slope peak and slightly increased noise after the grey dashed line are more likely related to transient effects after reaching the final capacity $K_{\bar{C}\bar{G}}$.

Varying the tuning of the coupling strength $K_{\bar{C}\bar{G}}$ above 50 tends to produce negative NBLE trends, while $K_{\bar{C}\bar{G}} < 50$ results in positive ones. These findings are not further investigated, but some thoughts about them are shared in the following. Firstly, changing the tuning of the capacity while keeping the total time interval fixed varies the rate of change of the $K_{\bar{C}\bar{G}}$, i.e., how fast the parameter is increased. Considering that the system exhibits a certain relaxation time to completely resynchronize after a disturbance, such as the parameter shift, this might explain the observation. If the capacity is increased slowly enough, the system follows quasi-static its fully synchronized state which progressively supports stronger synchrony by stronger coupling: The drift slopes decrease. If the rate of capacity change exceeds a critical time scale, the drift slopes indicate destabilization, since the system left the quasi-static stable branch, but still drifts towards it. In other words, it could be an issue of different time scales, similar to Supplementary Box S1 (but subcritical).

Secondly, it might also depend on the involved nonlinearity and the absolute capacities chosen, as they affect how strongly the slow signals of the nodes communicate and are fed into each other. There might be resonances for certain capacity levels, topologies, and more. On the one hand, the nodes may react more strongly to heavy frequency oscillations of individual nodes,

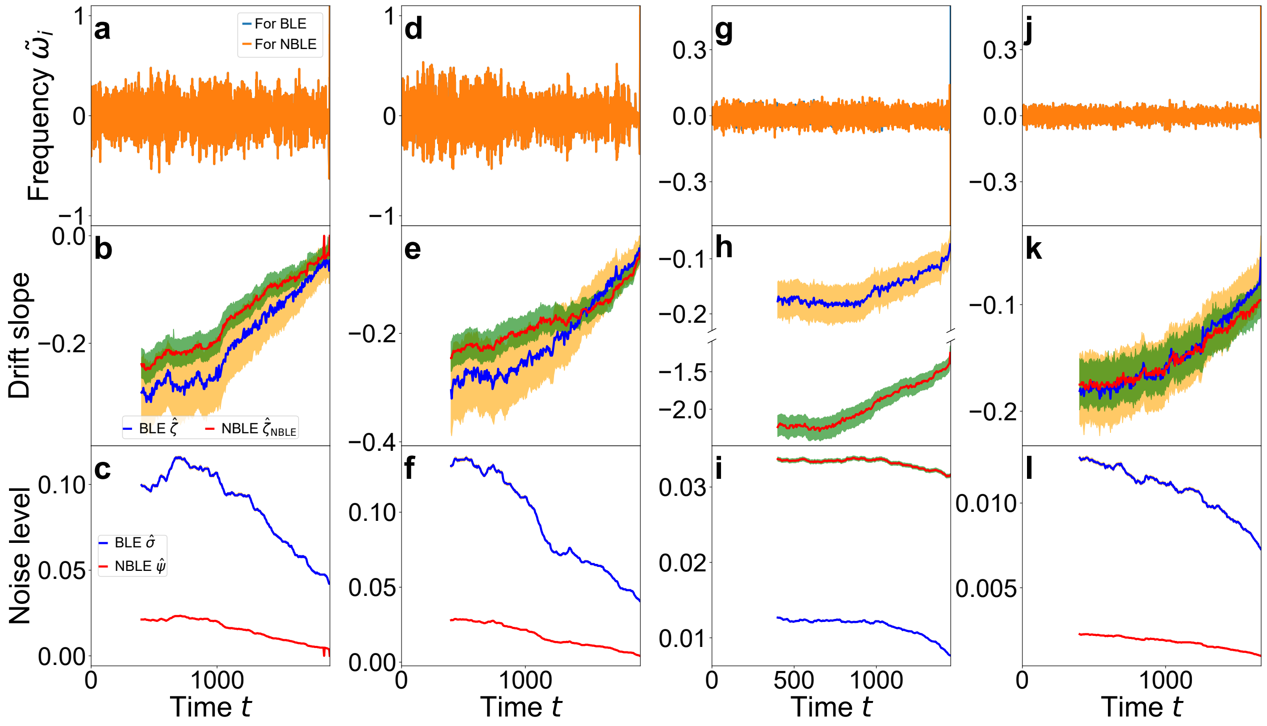

Figure S4. The real-world NAWI outage and the corresponding NAWI model signals in Figure S3 do not show the (N)BLE fingerprints of pure B-tipping on the CSE scale presented here. **(a–c)** B-tipping due to a critical linear decrease of the global grid capacity applied to all lines. Computations for ω_G are shown. Two windows did not converge at the end (spikes to zero). **(d–f)** Same for a local decrease of just one line capacity $K_{\bar{C}G}$. Results for ω_G are shown. **(g–i)** B-tipping due to global overload, i.e., a critical linear increase of the power across all nodes with $\sum_i P_i = 0$. Computations for ω_G (BLE) and $\omega_{\bar{C}}$ (NBLE) are shown. **(j–l)** Same for a local decrease of the power, still with $\sum_i P_i = 0$. Computations for ω_G are shown. The 16 % to 84 % CIs are shown for a comparison between BLE and NBLE. In all cases, the (N)BLE response shows increasing drift slopes $\hat{\zeta}_{(\mathrm{NBLE})}$, indicating B-tipping. Simultaneously, the fixed mesoscopic CSE noise appears to contribute less to the ASE dynamics, as evidenced by a relative noise decrease. Preliminary results suggest that for global parameter tuning, the (N)BLE does not strongly depend on the node from which the signal is taken. Small variations in the overall trend quality between signals from different nodes are observed. However, the signal location seems more important for local disturbances where the (N)BLE tends to show notably positive trends only in signals from nodes near the disturbance's periphery. In the NAWI model, these are signals from nodes that are affected by a line with decreasing capacity or by increasing power generation or demand. This corresponds to the Keeler-Allston line's nodes of Scenario (ii), which align with the McNary node G and the consumers $\bar{C}$ in Scenario (iii). These observations are preliminary. Further studies are needed to explore the behavior of (N)BLE in other topologies. For example, the current model only considers "dead end" generators, which have been suggested as vulnerable points for grid stability[16].

which might destabilize the frequency dynamics in a B-tipping scenario. On the other hand, strong coupling may favour better synchronization and stabilize the frequency if damping and noise levels are within a suitable range. These hypotheses require further investigation.
Finally, tuning capacities below a critical minimum destabilizes the system through B-tipping and results in a relative noise decrease (cf. Figure S4). If the grid's transmission capacity is diminished globally, the (N)BLE is similarly sensitive to frequency signals from all nodes. If only one line is affected, the (N)BLE response is stronger to signals of nearby nodes. In summary, the modelling attempts support the real-world observations of relationships between the components and the macroscopic frequency dynamics (cf. Results and Supplementary Box S1). Changes in parameters and characteristics of grid components, as seen in the Keeler-Allston line Scenario (ii), can modify the drift, including positive drift slope B-tipping trends, but also influence the noise propagation. The latter affects the relative macroscopic noise level of the ASE. Briefly, the dynamics of the key observable ω are reasonably related to the dynamics of the state $\underline{x}$ in Supplementary Box S1.

S3.3 The Loss of the McNary Power Units

Not least, we consider the loss of the McNary power units. When the system is perturbed from its stable operation regime, it is kicked to an alternative stable frequency due to a sudden load imbalance. The raw data of the NAWI frequency after the McNary loss exhibit a persistent frequency deviation of roughly $\sim 0.06\,\text{Hz}$, with a maximum of $\sim 0.13\,\text{Hz}$, which we do not replicate in our model. However, aligned with the real event, the scenario reproduces a jump to a new meta-stable frequency level where the ASE power balance assumption no longer holds.
Beyond the numerical artefact of one window length caused by the frequency spikes from the McNary loss, the (N)BLE drift slopes continue to indicate diminished local stability due to the load imbalance. Furthermore, the influence of power P_i on the deterministic part of the ASE is supported by the results in Figure S4(b,e). Tuning of the power to extreme levels that cannot be carried by the transmission capacities of the lines leads to destabilization via B-tipping. In addition, it tends to modify the relative macroscopic noise levels in a manner similar to B-tipping cases resulting from capacity tuning, as shown in Figures S4(c,f). Such destabilizing power levels might be realized on lower scales due to extreme electric demands, such as the intensive electricity trading involved in the NAWI failure. Similar to B-tipping caused by capacity tuning, global power changes elicited (N)BLE responses in all nodes, whereas a local power overload seems to yield (N)BLE fingerprints in nearby nodes. This might also depend on propagation times of the disturbance and the length of the simulations. This is left for future research projects.
In the shown simulation, the line to the McNary units is cut directly after the power loss. In a similar simulation (not shown), a case was considered in which the McNary power units' inertia is retained in the system for technical stabilization reasons and is cut only after power rebalancing. In this scenario, the jump in the drift slope due to the McNary power loss is slightly smaller, which may also be attributed to randomness in the computations. Moreover, it is hard to say whether cutting the line in the stabilized zone had any effect on the NBLE. A similar behaviour to that shown in Figures S3(i–l) was observed. Thus, it is likely related to transient effects following the primary control, i.e., increase of power $P_{\bar{G}}$ re-stabilized the frequency.
After the loss of the McNary units, power oscillations increased due to failure of the damping equipment. To isolate the influence of damping on the (N)BLE, the primary control action stabilizes the frequency again after the first red load imbalance interval in Figures S3(i–k). After the red dashed line, the damping is suddenly decreased, which leads to an upward trend in both (N)BLE drift slope and noise levels. The damping scenario that finally led to the islanding of the NAWI serves as a prototype case for Supplementary Box S2. In this scenario, the drawn analogy with connections between the one-dimensional frequency ω dynamics, the power grid's components, and the potential landscape of the power grid state $\underline{x}$ are particularly strong. Failing damping equipment flattens the stable potential valley, leading to lower local stability, as indicated by the drift slope. In the flattened potential, fast-scale fluctuations are less suppressed and, accordingly, the relative macroscopic noise level increases.

S3.4 Summary of the Results

Finally, the implications of the macroscale-mesoscale model comparison for the (N)BLE are summarized:

1. The power grid state $\underline{x}$ at different coarse-graining levels and the frequency ω are reasonably related, i.e., the frequency captures important aspects of lower-scale dynamics.

2. In the Keeler-Allston line example, integrating red instead of white noise reproduces the decoupling of BLE and NBLE drift slope estimates, as observed in the real time series. As expected, the NBLE better fits the $\mathrm{AC}(\tau)$ in Scenario (ii).

3. Tuning capacity and power in the mesoscale system, which are more closely related to microscopic components of power grids, affect the drift slopes and the relative noise level of the ASE and can lead to B-tipping, as illustrated in Supplementary Box S1.

4. Less damping creates positive trends in the drift slope and noise level, indicating a less stable state with increased dominance of mesoscale noise in the frequency dynamics. This is analogous to Supplementary Box S2.

5. If there is an effect from the actual line cutting in Scenarios (ii) and (iii) (grey dashed lines), it is too small to be resolved by the (N)BLE compared to other factors. The observed behaviour is more likely a transient effect of the recently reached dynamical states. However, cutting a line removes inertia from the grid, so it might be worthwhile to systematically consider such cases in the future.

6. Moreover, the results in Figure S4 suggest that local disturbances are predominantly detected by the (N)BLE in nearby nodes. These topological features are not examined in detail, leavin this for future studies.

7. During the computations, it was found that (N)BLE results can also depend on the rate of change regarding a specific parameter in the swing equation model. We suspect that this observation is related the ratio between the system's intrinsic relaxation time scale and the time scale of the parameter tuning, which ties into the basic mechanism of R-tipping (cf. Supplementary Box S3).

S4 Nonlinear Drift of the Bus Voltage Frequency

Real power grid frequency data tend to include nonlinear drift terms in their dynamics. Apart from other examples[20], also the pre-outage interval frequency $\omega_{\mathrm{P}}(t)$ tends to exhibit such nonlinear parts. In Figure S5, we show the drift for Segment 1 of Figure S13, since the data is assumed to be generated by an almost stable grid configuration. We denote the sum of squared errors by SSE. However, a first approximation of the frequency dynamics in stable operation by the ASE S.4 excludes higher-order terms of the empirically observed drift. Furthermore, it does not include alternative (meta-)stable frequency states for restoring rates $\bar{\gamma} > 0$, but diverges which is not observed in reality or less coarse-grained models as the CSE S.5. This observation further justifies the (N)BLE ansatz, which accounts for the nonlinearity of the drift by including it in the basic ASE approximation. The method approximates the unknown exact nonlinearity of the drift by its third-order Taylor expansion around the stable operation frequency.

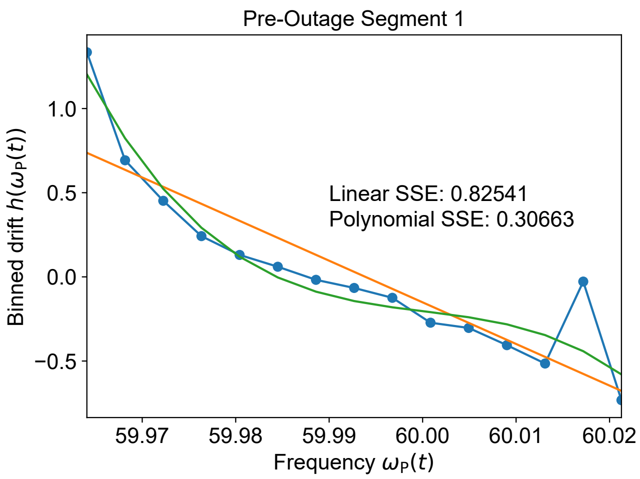

Figure S5. Real-world bus voltage frequencies in power grids are often better modelled by nonlinear drift functions. The pre-outage interval of the NAWI belongs to this class of frequency data. The sum of squared errors (SSE) of the third-order polynomial is more than twice as small than that of the linear fit. Another example is the frequency signal of the Ireland grid, which also exhibits nonlinear contributions in the drift function[20]. The blue curve shows the empirical drift of the data interval, averaged over fifteen bins. Briefly, the data increments are computed and assigned to the corresponding data points. Bins of these data are averaged and result in a non-zero drift function[21–23].

S5 Time Scales of Power Grid Disturbances and Primary Control

In this section, we briefly state individual regulative values for the grids of the Continental Europe Synchronous Area, taken from Ref.[24] (especially Annex III and Annex V therein):

- In general, global grid primary control actions in the Continental Europe synchronous area and related grids react to a maximum frequency deviation of ± 0.1 Hz to 0.5 Hz within a time frame from 10 s to 30 s.

- Global grid primary control actions in Germany react to a maximum frequency deviation of ± 0.2 Hz within up to 30 s. These actions must remain stable under full load for up to 15 min, after which the secondary control, simultaneously starting in regional grids, should fully replace the primary control[25–27]. This is consistent with the European Union regulations for the Continental Europe synchronous area.

- Furthermore, the regulation lists values of similar magnitude for related grids, such as the UK grid. The maximum steady-state frequency deviation for the Great Britain, the Ireland and Northern Ireland, and the Nordic grids is $\pm 0.5\,\text{Hz}$, whereby the Ireland and Northern Ireland grid regulation distinguishes between dynamical frequency deviations, set at $\pm 0.5\,\text{Hz}$, and static deviations, which are allowed up to $\pm 1\,\text{Hz}$.
- The activation times vary among different grids, with $10\,\text{s}$ for the Great Britain grid, $15\,\text{s}$ for the Ireland and Northern Ireland grid, and $30\,\text{s}$ for the Nordic grid.
- Unlike the activation times of the aforementioned grids, the activation time for the Nordic grid is defined from the moment when the standard frequency range $\pm 0.1\,\text{Hz}$ is crossed, which is smaller than the maximum steady-state frequency deviation.

S6 Bifurcation- and Noise-Induced Tipping

An illustrative introduction to the theoretical ideas behind modelling frequency dynamics via the Langevin approach is provided in Supplementary Box S1. The supplementary boxes aim to provide the reader with a rough idea of the system composition, the connection between grid components, grid state and the bus voltage frequency model, and tipping phenomena.
Typically, power grids (cf. Supplementary Box S1(a)) consist of various topologically structured levels, reaching from highest voltage grids, such as the transmissive supergrid of a country or continent, to local low-voltage grids, supplying cities and households with electricity. Additionally to the levels being connected to each other via electric tension transformation, there are different intra-level suppliers. On the one hand, these include nuclear or fossil power units and energy trading on the supergrid level, as well as on-shore wind parks or solar parks on the regional scale. On the other hand, there are various consumers, such as large-scale industries and railway on the high-voltage grid, and so on.
The grid components partially act on a controllable slow time scale $\tilde{\tau}_{\text{det}}$, which can thus be modelled in a deterministic fashion. The time scale is thought to coincide with the second to sub-second resolution of, e.g., trends in bus voltage frequency. The power grid's dynamics, influenced by these components, are basically modelled by the deterministic drift $\underline{h}(\underline{x}(t),t)$ which is related to the one-dimensional macroscopic frequency dynamics $h(\omega(t),t)$. We approximately assign the components that (partially) operate on a controllable time scale $\tilde{\tau}_{\text{det}}$ by a grey (grey-orange) tile in Supplementary Box S1(a). For example, this could involve the controlled (de-)activation of a power unit or industrial consumer, such as through demand response agreements. Nevertheless, industrial and scientific stakeholders only report activations and deactivations up to a certain extent and this might partially lead to stochastic fast-scale contributions.
Power outages typically evolve on time scales from seconds to minutes. Therefore, the daily periodic solar energy supply or the seasonal wind energy supply can be neglected, since they live on time scales much less relevant for power outage events. Accordingly, these factors should be compensated in the considered bus voltage frequency dynamics. In this spirit, the solar park example is mainly driven by additional phenomena, such as a variable cloudy and sunny sky, while wind parks are influenced by turbulent fluctuations in the atmosphere[13,28]. These components are therefore assigned by orange tiles denoting fast time scales.
The stable state $\underline{x}^*$ of a normally operating power grid is theoretically reflected by a valley in the potential $\underline{V}(\underline{x},t) = -\int \underline{h}(\underline{x}(t),t)\mathrm{d}\underline{x}$, with the mountainsides determining the strength of the restoring force through their steepness. The power grid states $\underline{x}$ are located in the coarse-grained Langevin framework. The frequency Langevin Equation 1 summarizes most of the information from less coarse-graind levels. A schematic illustration is given in Supplementary Box S1(b). Each available option of a control action and each infrastructure extension essentially affects the potential landscape. An unforeseen failure of a component can even transform the stable potential valley to an unstable mountain top, leading to a B-tipping event (cf. Supplementary Box S1(b)). Alternatively, it may decrease the height of the potential barrier, making N-tipping more probable (cf. Supplementary Box S2). All in all, if the dominant processes live on the time scale $\tilde{\tau}_{\text{det}}$, the PDF of the power grid states should be relatively peaked around the stable state, as shown in Supplementary Box S1(c). In this case, the fluctuations cannot overcome the potential barrier due to the restoring force in the potential landscape, as schematically shown by the black and green arrows in Supplementary Box S1(d). However, the grid is also composed of components that operate (partially) on a faster time scale, represented by orange (and grey-orange) tiles. These include the unpredictable usage of household end devices and smaller industrial machines (aside from simple trends such as the daily periodic usage of electric light, which are neglected in our scheme due to the significantly faster relevant time scales of power outages).
With respect to climate and environmental politics, societies around the globe advocate for upgrading power grids by employing renewable energy sources. The influence of faster time scales increases significantly through the integration of modern technologies. For example, wind and solar parks play a role at all grid levels. Combined heat and power units (CHPs), solar cells, energy storage technologies, and e-mobility are deployed at local grid scales. Essentially, the increasing intrinsic stochasticity could—in the hypothetical worst case—flatten the grid state PDF $p(x)$ for a given grid infrastructure (i.e., a specific potential landscape), as illustrated by the orange PDF in Supplementary Box S1(c). In such a scenario, intrinsic stochasticity can lead to

a power outage event due to N-tipping, as indicated by the orange arrow in Supplementary Box S1(d), because the restoring force cannot prevent the crossing of the barrier. In contrast, Gloe et al.[29] discuss how the inertia and primary control capabilities of wind turbines can help stabilize the electrical infrastructure.

The identified key factors that favoured the historic NAWI outage on 10th August 1996 are well captured by the aforementioned

Supplementary Box S1: The Langevin Model, pure B- and N-Tipping, and the Bus Voltage Frequency

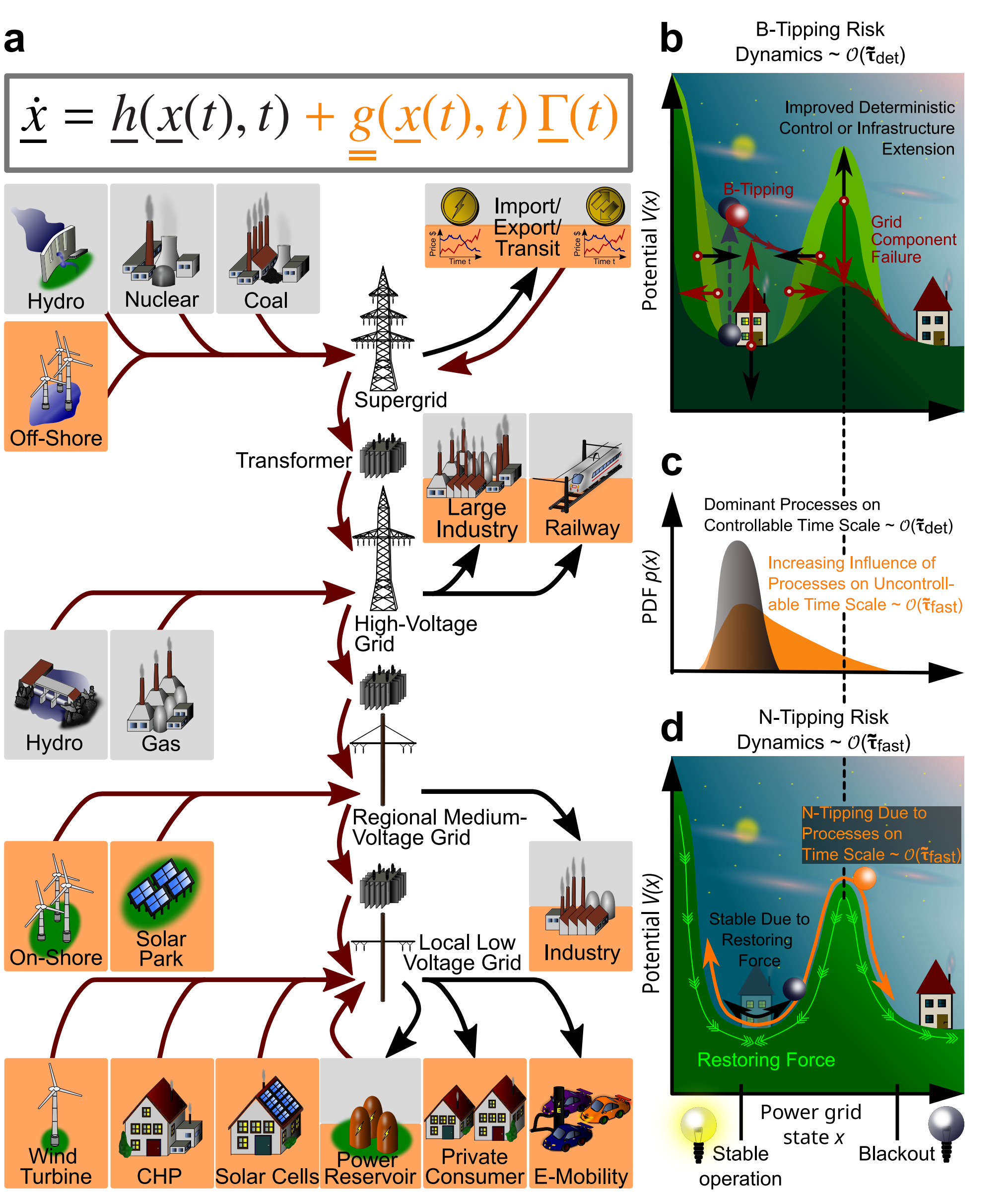

Relations between power grid dynamics, frequency dynamics, and the Langevin model. Prominent components are approximately assigned to each grid level as an example. **(a)** Power grids consist of various voltage levels from highest-voltage supergrid to low voltage local grid scales, connected by electric transformers. The grid suppliers and consumers operate on various time scales. Their dynamics can only be controlled and modelled by the deterministic drift $\underline{h}(\underline{x}(t),t)$ if they live on a suitable slow time scale $\tilde{\tau}_{\mathrm{det}}$. Contributions of the grid participants on shorter time scales $\tilde{\tau}_{\mathrm{fast}}$ are captured by the intrinsic stochastic dynamics $\underline{\underline{g}}(\underline{x}(t),t) \cdot \underline{\Gamma}(t)$. Operative time scales of the components are roughly assigned by grey, orange, and grey-orange tiles for $\tilde{\tau}_{\mathrm{det}}$, $\tilde{\tau}_{\mathrm{fast}}$, and mixed contributions, respectively[12,14]. **(b)** From a modeller's point of view, the power grid in stable operation is located at the minimum of the potential $\underline{V}(\underline{x},t) = -\int \underline{h}(\underline{x}(t),t)\mathrm{d}\underline{x}$. High-dimensional state representations $\underline{x}$ are not identical to the bus voltage frequency ω. A model's descriptive level and time resolution, i.e., its degree of coarse-graining, determine what is included in the drift and diffusion. Whereas the stable state $\underline{x}^*$ is a fixed point in this coarse-grained Langevin framework[30,31], the projection of the conceivable ensemble of power grid states into one dimension x is a simplification intended for illustration purposes. Nevertheless, the macroscopic frequency observable is indeed one-dimensional and consolidates most of the information about the fixed point region from less coarse-grained models (cf. SI S3). The landscape represents the current grid infrastructure. The system state can be envisioned as a ball influenced by stochastic kicks and the deterministic gravitational restoring force in the potential landscape. A component failure can alter the potential landscape by reducing the potential barrier, flattening it, or even flipping a stable potential valley into a mountain top or mountainside (shaded potential landscape) (B-tipping). **(c)** The presented PDFs do not resemble specific analysis results; rather, they illustrate the modelling concepts. If the power grid's control works properly, the PDF $p(x)$ of possible states is peaked around the stable operational state x^*, as illustrated by the grey-shaded PDF. Increasing influence of uncontrollable stochasticity—such as greater impact of orange-tiled fast-scale components in **(a)**—can widen the possible range of states, as indicated by the orange PDF. **(d)** If the restoring force (light green arrow line), given by the steepness of the potential mountainsides, is sufficiently strong, the power grid state fluctuates only slightly around the stable operational state (black arrows corresponding to the width of the grey PDF in **(c)**). Only when the restoring force is unable to compensate for increasing stochasticity does the probability for crossing the potential barrier rise (orange arrows) (N-tipping). For completeness, R-tipping is briefly discussed in SI S7.

Supplementary Box S2: N-Tipping by a Modified Potential

In addition to the scenario sketched in Supplementary Box S1(c,d), N-tipping can occur under stationary noise level if the potential barrier shrinks. This happens when the height of the potential maximum (potential landscape's mountain top) that separates the stable operational state from the blackout state decreases. For example, this may be due to grid component failures and decreasing stabilizing damping effects (cf. red arrows in Supplementary Box S1(b)).**(a)** The high potential barrier of the light green potential at time t_0 shrinks to the significantly lower barrier of the dark green potential, which features much flatter mountainsides at time t_1, as a result of a component failure at time t, with $t_0 < t < t_1$. In consequence, the restoring force is significantly decreased, making it much more likely to cross the weaker barrier after time t than before, even under the same noise level. This directly corresponds to a broadening of the PDF, but not due to changes in contributions on a time scale of $\mathscr{O}(\tilde{\tau}_{\text{fast}})$, as seen in Supplementary Box S1(c). Instead, referring to **(b)**, the grey PDF corresponds to the localized stable operational state in the light green potential valley at time t_0, which flattens to the orange PDF of the dark green potential at time t_1, although the noise level remains stationary in this example. For completeness, R-tipping is briefly discussed in SI S7.

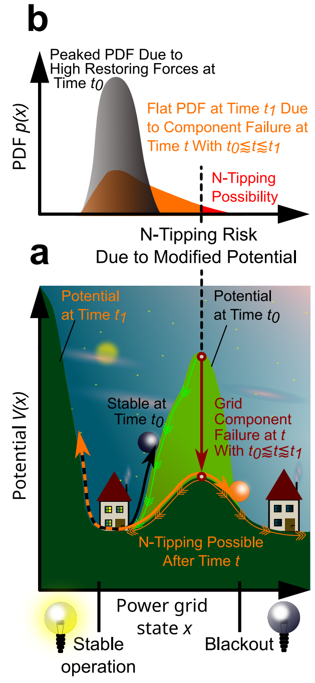

theoretical considerations. Although the influence of renewable energy sources was rather negligible in the 1990s (cf. "U.S. Energy System Factsheet" and "Renewable Energy Factsheet" of the University of Michigan[32,33]), the report of the power outage event identifies two contributors on a fast time scale that could potentially contribute to the diffusion $g(\omega(t),t)$ in the Langevin Equation 1: first, an unforeseen surge in energy demand from private and public consumers due to a heat wave, and second, increased energy exports driven by favourable conditions for hydroelectric power generation (even though still within established constraints)[12]. Both factors could contribute to a scenario as shown in Supplementary Box S1(c,d) or Supplementary Box S2. Furthermore, the report highlights several lapses in grid maintenance, such as the omission of trimming trees near power lines or disregarding the significantly reduced power supply from The Dalles hydroelectric power units, where only five of 22 were operating due to well-known protective measures for salmon migration during this period. The latter key factors directly contribute to the drift $h(\omega(t),t)$ in the Langevin Equation 1 and may have flattened the deterministic potential landscape, as illustrated by the red arrows in Supplementary Box S1(b).

S7 Rate-Dependent Tipping

In the main article, the focus lies on B- and N-tipping events. For completeness, a third typical tipping route, namely R-tipping, is also discussed in the context of power grids. In an R-tipping scenario, a system undergoes a transition that depends on the rate of change of a control parameter. In such cases, a critical transition may occur without explicit consideration of bifurcations or noise levels.

The phenomenon is sketched in Supplementary Box S3. During an R-tipping event, the potential landscape can preserve its fixed points. Nevertheless, it modifies so rapidly on a time scale of order $\mathscr{O}(\tilde{\tau}_{\text{shift}})$ that it surpasses the minimal time required for the system to respond to the changes driven by the potential's restoring force, which lives on a time scale of order $\mathscr{O}(\tilde{\tau}_{\text{relax}})$. Due to this discrepancy, if $\mathscr{O}(\tilde{\tau}_{\text{shift}}) << \mathscr{O}(\tilde{\tau}_{\text{relax}})$ is sufficiently satisfied, the system's state is suddenly relocated to another basin of attraction.

For power grids, this could correspond to a sudden and substantial failure of multiple control units. Even if the failure of the control units over a broader time interval would have been manageable for the system's stability, the simultaneous failure of all units in a very short time destabilizes the system through R-tipping. In principle, the BLE is related to the rate of change of a control parameter, as the time derivative $\dot{\zeta}$ of the drift slope estimates $\hat{\zeta}$ reflects the changes in the control parameter of the drift term in a way. Nevertheless, the phenomenon of R-tipping is currently beyond the reach of the method for several reasons. By definition, R-tipping is often poorly resolved, since the changes run off very fast. Consequently, the parameter changes must be discernible within the data collected per window, without producing a time delay due to the rolling window approach that exceeds the time scale of the R-tipping event itself. Furthermore, the relationships between the drift slope derivatives $\dot{\zeta}$, the control parameter change, and the system-dependent critical ratio $\tilde{\tau}_{\text{relax}}/\tilde{\tau}_{\text{shift}}$ are typically unknown.

Supplementary Box S3: R-Tipping Scenario in Power Grids

In a similar manner to Supplementary Boxes 1 and 2, the R-tipping mechanism is illustrated in the potential landscape. The grey PDF of the system state, shown in Figure **(a)**, corresponds to the accessible positions of the red ball in **(b)**, which is positioned within the light green potential at time t_0. As indicated by the black arrow, the ball is confined in the stable operation valley of its potential prior to time t. The confinement is governed by the restoring force (light green arrows), which corresponds to the steepness of the mountainsides. If the red ball is considered without any external changes, it would simply relax into the left valley with an intrinsic time scale of the order $\mathcal{O}(\tilde{\tau}_{\mathrm{relax}})$. However, in an R-tipping scenario the potential landscape is modified by a control parameter change that happens on a much faster time scale of the order $\mathcal{O}(\tilde{\tau}_{\mathrm{shift}})$. As a result, the grey PDF in **a** approximately remains stationary. In this sense, the specific ratio of $\mathcal{O}(\tilde{\tau}_{\mathrm{shift}}) << \mathcal{O}(\tilde{\tau}_{\mathrm{relax}})$ is crucial for the R-tipping mechanism. Even if the stable and unstable fixed points are retained, they are shifted so rapidly that the red ball cannot follow the shifted light green potential on a time scale comparable to $\mathcal{O}(\tilde{\tau}_{\mathrm{shift}})$. This is due to the restoring force, driving the ball on a slower time scale $\sim \mathcal{O}(\tilde{\tau}_{\mathrm{relax}})$. Instead, the potential landscape beneath the ball, which remains almost stationary on the much faster time scale $\tilde{\tau}_{\mathrm{shift}}$, changes to the shaded dark green potential. In consequence, the red ball experiences the new restoring force that guides it to relax into the blackout state at time t_1, shortly after the shifting time t. In a power grid, this could occur due to a sudden and significant failure of multiple control units, making it impossible to maintain a stable operational state. Under normal circumstances, the remaining control units could stabilize the grid if the failures had occurred gradually over time instead of all at once.

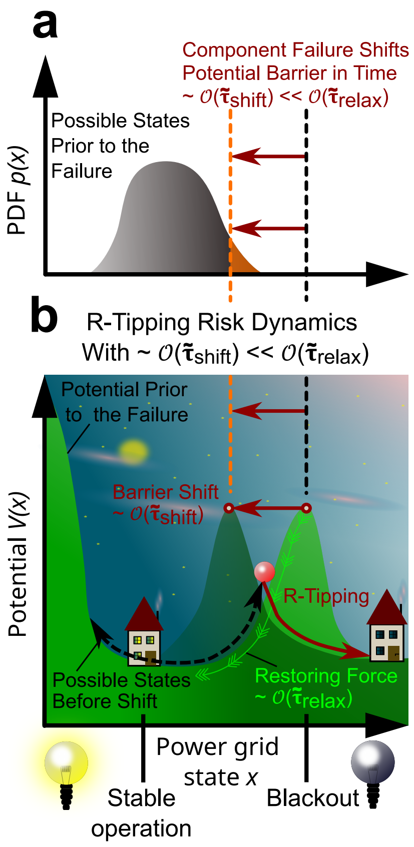

S8 Details of the Western Interconnection Cascading Failure

The information is mainly drawn from the Western Systems Coordinating Council's (WSCC at that time; now WECC) approved “Disturbance Report”[17] and the “1996 System Disturbances” report[34] from the North American Electric Reliability Council (NERC), but cf. also Refs.[35–39] and Appendices of the “Preliminary Disturbance Report”[40].

At 14:06 PDT[1], the 500 kV Big Eddy-Ostrander line was opened by system protection because it flashed and grounded to a tree, which led to several pre-outage line openings. These events led to a significantly less resilient power grid state. Under these circumstances, the THIF[18,19] of the 500 kV Keeler-Allston line had heavy impact on the grid stability: In the approved disturbance report[17], it is identified as the key triggering event of the NAWI cascading failure on 10th August 1996.

What followed was an interplay of control actions, component failures due to inadequate maintenance, human errors, culminating in a further cascade of various line openings. The events were compensated by increasing the reactive power of the McNary units to a maximum, which necessitated their removal from service between 15:47:40 and 15:47:52. This marked the beginning of mild power and voltage oscillations in the transmission system, which increased over time. As a direct consequence, the entire grid was split into four isolated subgrid islands: the Northern, the Northern California, the Southern California, and the Alberta islands. This major power outage interrupted electric service to about 7.5 million customers, the last of whom had their electric service restored around 1:00 on 11th August 1996.

Kosterev et al.[38] identified the power grid disturbance as being caused by a so-called *small-signal instability*: The NAWI grid was known for damped interarea oscillations with a frequency around 0.2 Hz to 0.3 Hz, which were typical of the grid's topology. This topology made it vulnerable to oscillations among certain machine clusters against others due to weak interconnections. If some control actions failed or the grid topology was unexpectedly modified, the damping could no longer stabilize the system. As a result, the small signal disturbances were able to persist and grow, ultimately leading to the power outage.

[1] All times in this work are given in the local Pacific Daylight Time (PDT).

S9 Limits of the First Approximation: The Parametric Ornstein-Uhlenbeck Estimation

When a complex system approaches a bifurcation, this can be accompanied by a decreasing restoring rate, and conversely, by an increasing relaxation time. This CSD is the starting point of non-parametric leading indicator argumentations: An equidistantly sampled time series should exhibit positive trends in AR1 $\hat{\rho}_1$ and STD $\tilde{\sigma}$ over time because perturbations persist longer if the restoring rate decreases due to CSD. However, since the limitations of such non-parametric reasoning are already discussed, we focus here on a common variant of parametric reasoning that involves the statistical measures AR1 $\hat{\rho}_1$ and STD $\tilde{\sigma}$. The parametric approach approximates the data-generating process as an OU process

$$\dot{x} = \zeta_{\mathrm{OU}} \cdot x + \sigma_{\mathrm{OU}} \Gamma(t),$$

i.e., a Langevin equation with a linear drift $h(x) = \zeta_{\mathrm{OU}} \cdot x$, constant diffusion $g(x) = \text{const.} \equiv \sigma_{\mathrm{OU}}$, and a Gaussian δ-correlated noise process $\Gamma(t)$. The AR1 ρ_1 and STD $\tilde{\sigma}$ of an OU process realisation is given by

$$\begin{aligned} \rho_1 &= \exp(\zeta_{\mathrm{OU}} \Delta t) \\ \tilde{\sigma}^2 &= -\frac{\sigma_{\mathrm{OU}}^2}{2\zeta_{\mathrm{OU}}} \end{aligned} \tag{S.7}$$

with the discrete time step Δt.[41] Note that the non-parametric leading indicators implicitly build on such dependencies $\rho_1(\zeta_{\mathrm{OU}})$ and $\tilde{\sigma}^2(\zeta_{\mathrm{OU}})$, but do not necessarily parameterise a specific model. For pure B-tipping, all parameters of Equations S.7, apart from the restoring force, are assumed to be constant. Let us exemplarily illustrate this by using the OU model with restoring rate ζ_{OU} (which is not necessarily specified in the non-parametric ansatz). Emerging CSD can be described as follows:

$$\begin{aligned} &\lim_{\zeta_{\mathrm{OU}} \to 0} \rho_1(\zeta_{\mathrm{OU}}) = 1, \qquad \text{indicating a positive trend } \rho_1 \nearrow \\ &\lim_{\zeta_{\mathrm{OU}} \to 0} \tilde{\sigma}^2(\zeta_{\mathrm{OU}}) \to \infty, \qquad \text{indicating a positive trend } \tilde{\sigma}^2 \nearrow . \end{aligned}$$

However, in the parametric ansatz, we explicitly define the OU model to approximate the data-generating process. That means, once the AR1 $\hat{\rho}_1$ and STD $\tilde{\sigma}$ of a time series are estimated in rolling windows, they can be transformed by Relations S.7 into the OU restoring rate estimates $\hat{\zeta}_{\mathrm{OU}}$ and the noise level estimates $\hat{\sigma}_{\mathrm{OU}}$ by

$$\begin{aligned} \hat{\zeta}_{\mathrm{OU}} &= \frac{\log(\hat{\rho}_1)}{\Delta t} \\ \hat{\sigma}_{\mathrm{OU}} &= \sqrt{-2\tilde{\sigma}^2 \hat{\zeta}_{\mathrm{OU}}} = \sqrt{\frac{-2\tilde{\sigma}^2 \log(\hat{\rho}_1)}{\Delta t}}. \end{aligned} \tag{S.8}$$

Since the autocorrelation function $\rho_{\mathrm{OU}}(\tau)$ of an OU process satisfies $\rho(\tau) > 0$ for all time lags τ, the term $\log(\hat{\rho}_1)$—which enters in both quantities of Equations S.8—is only defined as long as the OU approximation is sufficiently met. Generally, for time series of arbitrary processes it holds $\rho(\tau) \in [-1, 1]$.

We apply the alternative OU estimation (OUE) procedure to the synthetic datasets from Figure S1. In Figure S6, these estimates $\hat{\zeta}_{\mathrm{OU}}$ and $\hat{\sigma}_{\mathrm{OU}}$ are compared to the BLE estimates $\hat{\zeta}$ and $\hat{\sigma}$, as well as to the ground truths, indicated by blue and red dotted lines, respectively. In all cases, the OU estimates are strongly biased, with deviations up to roughly a factor of five compared to the ground truth, before they diverge to $\pm\infty$. This divergence is caused by the above-mentioned dependency on $\log(\hat{\rho}_1)$ in combination with occasionally negative AR1 $\hat{\rho}_1$. Besides, in Figures S6(e,g,h), the OU noise level estimates $\hat{\sigma}_{\mathrm{OU}}$ exhibit trends which contradict the constants and trend directions of the ground truths.

Note that also for the purely B-tipping fold scenario in Figure S6(c,g), which is a prototype example of a critical transition, the divergence of the OU estimates is observed. Only in the case of the purely B-tipping pitchfork model in Figures S6(a,e), does the OUE provide stable estimates across the entire range, albeit strongly biased. In contrast, the BLE yields stable and unbiased results in all scenarios with almost perfect congruence to the ground truths.

In Figure S7, we demonstrate that these implications are also valid for practical examples, such as real-world melt rates from the Greenland Ice Sheet. For a more detailed discussion on the analyses of the empirical time series from Western Central Greenland (CWG) and the Nuussuaq (NU) peninsula, see Ref.[42].

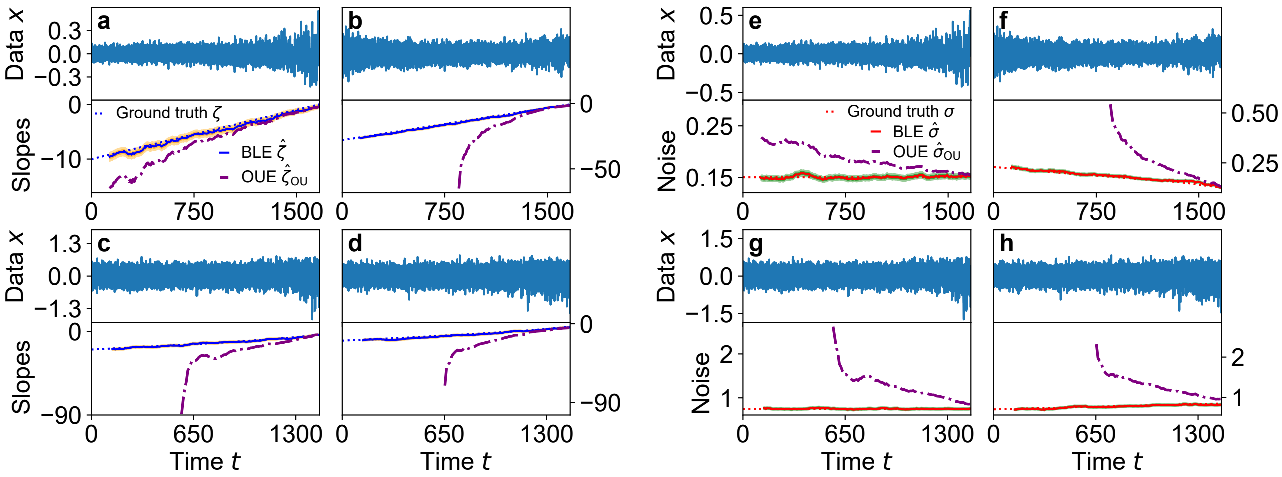

Figure S6. Comparison between the parametric BLE and OUE estimates to the ground truths of the four synthetic datasets that are previously introduced in Figure S1. **(b–d,f–h)** The OUE, which can be seen as a first approximation to the data, breaks down for intervals with $\hat{\rho}_1 < 0$, which leads to diverging estimates $\zeta_{\mathrm{OU}} \rightarrow -\infty$ and $\sigma_{\mathrm{OU}} \rightarrow \infty$. In general, the estimates are strongly biased. **(e,g,h)** In particular, the noise estimates σ_{OU} contradict the trends of the ground truths. Note that the divergence and strong bias also occur for the purely B-tipping fold model in **(c,g)**, which can be seen as prototype of catastrophic transitions. In contrast, the BLE results are stable and unbiased over the entire time interval.

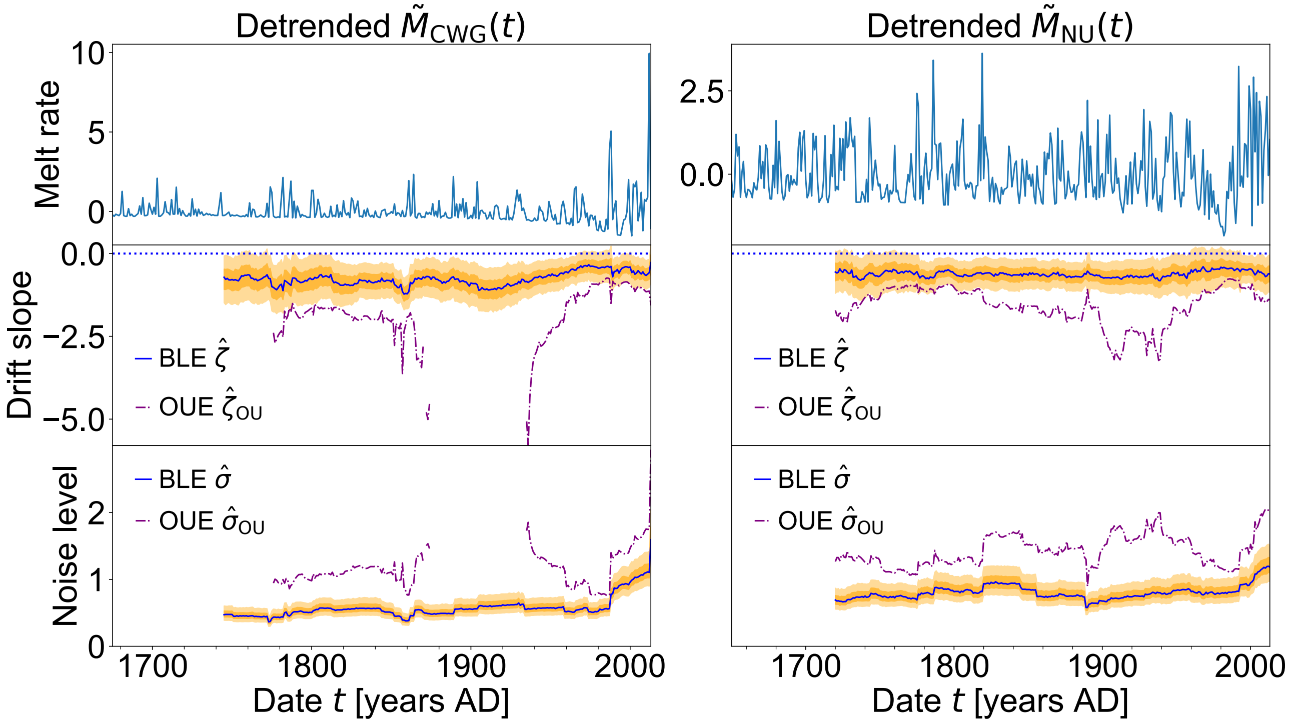

Figure S7. The comparison between OUE and BLE confirm the superiority of the BLE over the OUE, previously observed for synthetic data, for a prominent real-world climate science example. A comprehensive discussion of these evaluations for potential tipping points in the melt rate dynamics of the Greenland Ice Sheet in CWG and the NU peninsula are left for Ref.[42].

S10 The Bayesian Langevin Estimation in a Living System

In this section, a dataset from real-world population dynamics is evaluated to demonstrate the wide applicability of the BLE. In 2011, Veraart et al.[43] performed a "population collapse" experiment with *Aphanizomenon flos-aquae*. The population dynamics of these cyanobacteria depend on the incoming light and are well understood: On the one hand, light is needed for photosynthesis. On the other hand, too much light negatively affects the bacteria culture's growth. Under stable conditions, the cyanobacteria provides the needed mutual shading to protect against excessive light stress to support its own growth. This establishes a positive feedback mechanism and causes the bistability of the living system. The population is measured in terms of light attenuation over more than 25 d, as shown in Figure S8. The data is freely available[43,44]. Originally, the experiment was designed to directly extract recovery rates from the appropriately performed dilution of the bacteria culture. These events correspond to the six abrupt decreases of the light attenuation. Two hours before every dilution event, the light intensity was increased to provoke destabilization due to a fold bifurcation after roughly 25 d. Since the cyanobacteria's local stability shall be estimated with the BLE, the focus lies on the analysis of data segments before dilution to guarantee that the system is approximately in its stable equilibrium. The analysed segments are indicated in Figure S8.

For each segment, the BLE is performed on windows of size $N_\mathrm{w} = 200$ data points, using a shift of one point per window. The approach to show the significant positive trend in the drift slope estimates is similar to the procedure applied by Grziwotz et al.[44]. The BLE results of the shortest Segment 2 yield 57 correlated estimates. Therefore, only the last 57 drift slope estimates of each segment are used. These data subsets are collected immediately before the subsequent dilution event and consistently include the increase in light stress, making them the most comparable. Every sixth value is used, resulting in 10 estimates per segment. This approach partially accounts for the serial correlation introduced by the overlapping rolling windows. Note, however, that the estimates from one segment to the next are uncorrelated. This is more important because the inter-segment, rather than the intra-segment, trend is considered. The results are presented in Figure S9. The significance is first tested in the Bayesian framework using BFs[45], and second, in a frequentist hypothesis test using the Python ordinary least squares fit function *statsmodels.api.OLS(...)*[46]. The frequentist approach tests against the hypothesis $\mathscr{H}_0$ that the data can be explained by a constant.

In contrast, the BFs

$$\mathrm{BF}_{ij} = \frac{p(\hat{\zeta}|\mathscr{M}_i)}{p(\hat{\zeta}|\mathscr{M}_j)} \text{ with } i,j \in \{1,2\} \text{ and } i \neq j \tag{S.9}$$

represent the odds ratio of the probabilities that a linear positive gradient model

$$\mathscr{M}_1 : \hat{\zeta} = f_1(t) = b_0 + b_1 \cdot t \tag{S.10}$$

or a constant model

$$\mathscr{M}_2 : \hat{\zeta} = f_2(t) = \text{const.} \equiv \mathrm{b}_0 \tag{S.11}$$

explain the computed drift slope data $\hat{\zeta}$. Therefore, 10^5 model parameter tuples are drawn in a Monte Carlo experiment from the prior distributions

$$p(b_0) = \mathscr{N}(\mu = \langle\hat{\zeta}\rangle, \sigma = 5), \tag{S.12}$$
$$p(b_1) = \mathscr{U}(0.5, 1.5), \tag{S.13}$$
$$p(\log(b_2)) = \mathscr{U}(\log(x_\mathrm{min}), \log(x_\mathrm{max})) \tag{S.14}$$

where b_2^2 denotes the variance of the residual uncertainties, $\langle\hat{\zeta}\rangle$ the mean drift slope, and $\mathscr{U}$ a uniform distribution. The limits $\log(x_\mathrm{min})$ and $\log(x_\mathrm{max})$ are adjusted in each test case to include the residual variances from both models. The BFs thus quantify the degree to which one specified model is preferred to the other. However, note that the used homoscedastic likelihood function

$$p(\hat{\zeta}|\mathscr{M}_i) = \int \prod_i \frac{1}{b_2\sqrt{2\pi}} \exp\left(\frac{(\hat{\zeta} - f_i(t))^2}{2b_2^2}\right) p(\underline{b}|\mathscr{M}_i) \tag{S.15}$$

assumes uncorrelated measurements which does not hold for the intra-segment data. For the overlapping window approach, the BF is therefore biased and must be interpreted in relative rather than absolute favour of one model over the other. For example, with $\mathrm{BF}_{12} = 1000$, it cannot be concluded that model $\mathscr{M}_1$ is 1000 times more probable than $\mathscr{M}_2$, but it is definitely a better description of the measured data. Nevertheless, the BFs and frequentist statistics on the BLE results are also computed for two non-overlapping window runs with sizes $N_\mathrm{w} = \{50, 100\}$, which avoid the correlation biases introduced by overlapping rolling

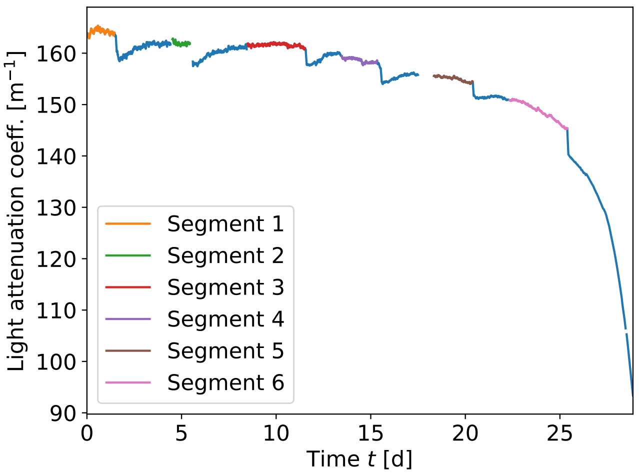

Figure S8. The cyanobacteria population size over time in terms of the light attenuation coefficient. The gradually increasing intensity of incoming light over roughly 25 d leads to a population collapse described by a fold bifurcation. Six abrupt decreases in population size are due to dilution events performed for a direct calculation of recovery rates[43]. These events are excluded from the analysis with the BLE, which is suited for unperturbed population dynamics. For this reason, similar to Grziwotz et al.[44], we restrict the analysis to the indicated data segments before the dilution events. These segments approximately correspond to the unperturbed population dynamics under almost comparable experimental conditions.

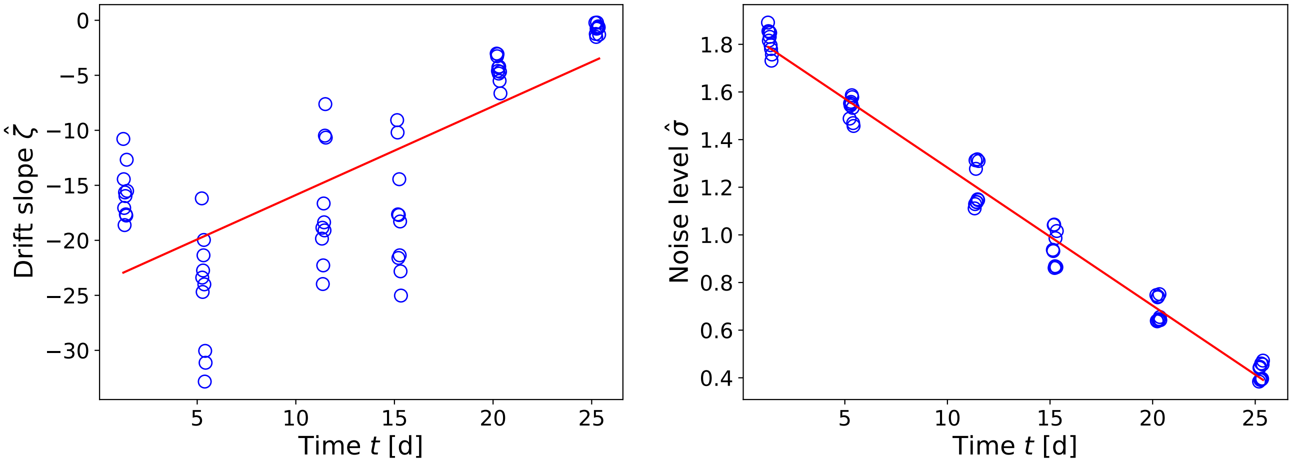

Figure S9. BLE results of the "population collapse" experiment. The linear ordinary least squares fits are computed using every sixth estimate of the last 57 data points per segment, accounting for the unequal lengths of the segments (cf. Figure S8) and partially addressing the intra-segment correlations due to the overlapping rolling windows. There is a significant positive trend in the drift slopes $\hat{\zeta}$, indicating decreasing local stability of the living cyanobacteria population. The noise level estimates $\hat{\sigma}$ are linearly decreasing which correlates with the reduction in measurement noise, previously discussed in the original article by Veraart et al.[43]. For more information, the reader is referred to the main text.

windows. Unbiased, a $\mathrm{BF}_{12} > 20$ commonly means very strong evidence in favour of model $\mathscr{M}_1$ over $\mathscr{M}_2$. The described statistics are computed on the centred drift slopes $\hat{\zeta} - \langle\hat{\zeta}\rangle$ plotted against the shifted time stamps $t - \langle t\rangle$, resulting in $b_0 \approx 0$. This transformation facilitates the convergence of the BFs with respect to the intercept prior and focuses the significance tests on the gradient. The results of the significance test are summarized in Table S3.

Finally, the decreasing noise levels suggested by the BLE in Figure S9 are briefly discussed. In the original article[43], it is documented that the experimental framework results in a decreasing measurement noise of the vertical light attenuation due to the Lambert-Beer law if the incoming light intensity is increased. This effect is captured by the decreasing BLE noise levels. Furthermore, the data exhibit almost no intrinsic stochasticity, which contradicts the Langevin model ansatz. The results confirm that the BLE, on its own, cannot distinguish between measurement noise and intrinsic stochastic contributions. This must be kept in mind when the intrinsic noise and the measurement noise are expected to be of similar magnitude, as the BLE results are likely most affected in such cases. Otherwise, for a proper interpretation of the estimated noise levels, it is worth knowing about the relative strengths of both contributions. In this experiment, almost no intrinsic stochastic variations are expected and thus, the measurement noise is dominant in the noise level estimates.

We emphasize that the BLE is not only applicable to the real-world dynamics of living bacteria cultures and power grids. Quite the opposite, the BLE is a general time series analysis algorithm that is applicable across various disciplines. For example, it provides deeper insights into theoretical food web dynamics in ecology[8], the greenhouse-icehouse transition in paleoclimatology[47], the Greenland Ice Sheet in climate science[42], meta-stable market states in economy[48], and more.

Window Size	Overlap (Window Shift)	OLS Slope b_1	BF_{12}	p	R^2
200	True (1)	$8.07 \cdot 10^{-1} \pm 9.7 \cdot 10^{-2}$	$3.5 \cdot 10^{9}$	$p < 0.001$	0.545
50	False (50)	1.228 ± 0.359	$1.8 \cdot 10^{2}$	0.001	0.151
100	False (100)	0.980 ± 0.375	23	0.014	0.186

Table S3. Significance tests for a positive gradient in the drift slopes $\hat{\zeta}$ of the microcosm experiment with cyanobacteria. The first row corresponds to the statistics of the drift slope analysis shown in Figure S9. Note that the BF in the example with overlapping windows is biased and therefore the results have to be interpreted with caution. Keeping that in mind, the BF suggests a preference for a non-zero gradient model. The frequentist approach with p-value and coefficient of determination R^2 lead to the same conclusion. Additionally, the statistics of two analyses without overlapping windows are computed to avoid the bias of serial correlations. The results are significant regardless of the approach, although the explanatory value in terms of R^2 is notably smaller for the non-overlapping windows. This might be due to the serial correlations present in the overlapping window analysis, as well as the need for smaller window sizes in the non-overlapping analyses due to the shorter data segment lengths. The smaller window sizes lead to heavier fluctuations of the estimates. The uniform prior ranges $[\log(5), \log(10)]$, $[\log(20), \log(25)]$, and $[\log(13), \log(18)]$ for $\log(b_2)$ are used to compute the BFs corresponding to the window sizes $\{200, 50, 100\}$, respectively.

S11 Metadata Research

The two time series of the North America Western Interconnection (NAWI) blackout on 10th August 1996, which we analyse in the Results section, were originally provided without corresponding metadata, i.e., absolute times and measurement locations. Available information only claimed both time series to be recorded with similar time resolution directly prior to the outage event[49–51]. We wondered about the significantly differing frequency measurements, since they were suggested to stem from the same period in time, covering the same line of events. Since the power grid frequency is a macroscopic key observable of AC power systems, being the same across all nodes, we expected to observe at least similar time evolution prior to the outage. Due to this discrepancy, two main hypotheses emerged:

1. Both time series might have indeed covered the same time interval, but were measured at different locations. Following this idea, the differing frequency time evolutions could be attributed to topological features of the NAWI or to different islands that formed during the cascading event.

2. If the first hypothesis were not true, the time series may have been incorrectly reported as covering the same time period. Even if they both might correspond to the NAWI cascading failure on 10th August 1996, they could refer to distinct stages in the outage cascade.

Furthermore, regardless of the actual outcome of the source's approval, it seemed to be an interesting project to dive into a detailed comparison between the analysis results and the real timeline of the historic blackout event. A check up of the data sources was necessary to dispel our doubts. The search spans roughly 1.5 a including intense correspondence with four institutions, i.e., Bonneville Power Administration (BPA) via the Freedom of Information Act (FOIA) (correspondence with James King (FOIA Public Liaison, BPA) and Brian Roth (FOIA Case Coordinator, BPA)), WECC, the Washington State Library of the Office of the Secretary of State, Steve Hobbs, (correspondence with Mary Schaff (Librarian, Washington State Library) via the "Ask a Librarian" Service) and the Seattle Municipal Archives (correspondence with Jeanie Fisher (Reference Archivist, Seattle Municipal Archives)).
At the beginning, we were provided with several data files from BPA which might have included the two time series and hopefully the missing metadata. Indeed, we could identify the pre-outage frequency record $\omega_P(t)$ (cf. main article, Figure 1) in one of the files. In further correspondence with BPA employees, we could clarify that the record is measured in Tacoma and starts at 15:29:40 o'clock on 10th August 1996.
Unfortunately, the second time series shown in Figure S10(a) was not amongst the provided data files. Furthermore, we got the information that BPA measured frequency only in Tacoma and Dittmer at that time. Since Tacoma and Dittmer both belong to the Northern grid island and are separated by only $\sim 220\,\text{km}$, the new information largely excluded Hypothesis 1. At this point, Hypothesis 2 or some related error in the time scale became more likely. Since we knew that the second time series was

digitized from a frequency time series scan from the Western Systems Coordinating Council's "Disturbance Report"[17,40] by other researchers[50,51] in the field via a software tool, we reached out to them in order to receive the original scan, hoping to find notes about date and absolute times of the second dataset therein. We are grateful for the fast reply and the support of Professor Eduardo Cotilla-Sanchez. Unfortunately, he was not able to provide the original source, since the authors only got access to the printed responsive reports for a limited time period in 2012. However, he subsequently supported the search for the data source by valuable suggestions and discussions.
Thanks to a further BPA FOIA request we got the necessary reports. Although the version contains the List of Exhibits, it does not contain the actual figures and tables. Therefore, we reached out to WECC, which was finally able to provide us with four pages of a frequency plot from the disturbance report. Unfortunately, the scan is of very low quality and the time axis is not readable. However, after carefully comparing the scan to our digitized version in Figure S10 (a), we noticed that it matched up apart from some positive offset of the digitized frequency values on the right. We assumed that to be caused by the data extraction procedure from the low quality scan. Nevertheless, it nourished further doubt about the originally stated metadata: Based on these, the high peak of the frequencies in Figure S10 (a) was declared to be the beginning of the outage event. The comparison to the low quality scan version showed that the frequencies in the black-hatched area are artificially lifted by the data extraction procedure and should line up at 60 Hz. Against this background, the originally stated metadata would have implied that the frequencies before the outage's peaks were less stable (flickered stronger around 60 Hz) than directly after the peak, when the blackout event already occurred. Furthermore, this stable frequency region would have been reached within seconds to a few minutes, though power supply to all customers was gradually restored over several hours until the next day, and the grid was completely restored over the course of days, concluding on 16th August 1996[17].
Subsequently, we identified the figure description of Exhibit 10 in the disturbance report to correspond to the low quality scan. This enabled us to draft a more precise request to the Washington State Library via the "Ask a Librarian" service. Since Mary Schaff had only access to a scan of similarly disappointing quality in the Washington State Library, we asked her for alternative institutions which would hopefully archive a better resolved version. Finally, Mary Schaff reached out to several colleagues and got positive response from Jeanie Fisher at the Seattle Municipal Archives. We are very grateful for the dedicated help of all the people who participated in solving the puzzle of the lost data source. Additionally, Mary Schaff let us have the timeline tables of the approved report's Exhibits, i.e., Exhibits two, three, five and nine, and Jeanie Fisher sent us a full version of the preliminary report.
Unexpectedly, the readable time axis is labelled in daytime hours and disagrees significantly with the originally[50,51] reported one. It spans almost a period of 20 h instead of 10 min of the *post*-outage period instead of the *pre*-outage period and time runs from right to left instead of vice versa. Since this strongly disagrees with the previously assumed setting, we tried to find notes in the preliminary and approved report about time scaling errors present in the frequency time series, but without success. Therefore, we summarize the arguments for the time scale assumed in the Results section in the following list.

Major Arguments:

1. Finally, the original source with readable time and frequency axes is accessible. The axes significantly differ from the originally assumed and reported[49,51] setting. A search for notes about axis labelling errors in the approved and preliminary report was not successful. Furthermore, detailed information regarding the time scales, absolute times, and measurement locations for nine additional exhibits, which contain 63 more data plots, are consistently provided. The time axis runs from right to left, which indicates that the frequency time series, used in previous articles[44,49–51] and shown in Figure S10(a), should be inverted. Furthermore, the time series spans mostly the *post*-outage interval instead of the pre-outage period, covers roughly 20 h instead of 10 min, and is sampled 265 times less dense than expected from the originally reported dataset[50,51].

2. The documented time scaling in the disturbance report exactly fits the time stamps of real events. Fore example, the first high peak is observed around the first islanding process at 15:49. In particular, after 15:49, "the frequency stayed high in the Northern Island (about 60.4 Hz for 14 minutes, crossing 60 Hz after 17 minutes, dipping as low as 59.95 Hz, then rising to 60.04 Hz for the next 50 minutes)"[17]. The easier available NERC report describes the frequency time evolution as follows: "The North island frequency rose to 60.9 Hz dropping to 60.4 Hz within two seconds where it remained for about 14 minutes. The frequency crossed 60 Hz three minutes later"[34]. Both descriptions perfectly fit the frequency time series assuming the scaling as stated in the approved disturbance report.

3. Based on the time scaling, documented in the approved report[17], it was tried to align the presumed pre-outage segment (cf. black-hatched interval in Figure S10(a)) of the time series with frequency time series provided by BPA, for which the absolute times and the measurement location are known. The alignment suggests that the time series indeed correspond to a record of roughly 20 h, primarily in the post-outage region. For more information about the alignment procedure, the interested reader is referred to SI S12.

4. The inverted time direction is further suggested by features of the time series when interpreted from right to left: Under this assumption, the pre-outage frequencies are well-located around the expected stable 60 Hz and fluctuate more strongly after the main blackout events, indicating a less resilient and less stable power grid state. Besides, the rather sudden separation of the islands is well-reflected by the sudden peak to roughly 60.4 Hz which relaxes towards 60 Hz again, instead of increasing further in a sawtooth pattern, as the originally proposed time direction would suggest.

5. The originally assumed time stamps seem to be highly unlikely considering that the corresponding time series, shown in Figure S10(a), would indicate completely different frequency dynamics than those in the time series approved by BPA.

In addition to these main arguments focusing on technical and empirical details of the time series in question, there are additional points that can be summarized as minor arguments.

Minor Arguments:

1. It is likely that the time series scan of the approved report[17] originates from a measuring device that printed the measured values directly onto scale paper via a writing arm. Since the scale paper's axes are fixed, the printing direction, i.e., the time direction, is defined by the scale paper used and by the calibration of the measuring device. This may explain why the time axis runs from right to left, rather than the typical Western reading direction from left to right.

2. BPA did not locate a data record corresponding to the request for pre-outage data. This may be attributed to the measurement not being stored in digital form but only printed, as mentioned above. Alternatively, it could be due to the initial request being based on the incorrect assumption of searching for time series data, as presented in Figure S10(a), *prior* to the outage at sub-second resolution, rather than mostly *after* the outage at second resolution.

Details of the time scale reconstruction are documented in SI S12.

S12 Time Scale Reconstruction of the Post-Outage Frequency Time Series

In Section S11, the intensive research for the source of the post-outage frequency time series, the analysis of which is presented in the Results section, is summarized. After about 1.5 a, we finally found evidence of mistakes in the originally reported time scale in previous articles[44,49–51]. The reasons for these errors are most likely twofold and closely related to the fact that it was digitized via a software package from a printed time series scan:

1. In most of the available sources, the time series scan is only provided in remarkably bad quality that impedes reading the time axis. Therefore, the time axis was presumably interpreted in the common Western reading sense, i.e., from left to right (cf. Figure S10 (a)), instead of vice versa, which would have been correct for this scan. In addition, frequency peaks of 60.4 Hz occur at the erroneously defined "end" of the time series. This was most likely misinterpreted as the beginning of the outage, leading to the fallacy that the time series scan predominantly covers the pre-outage region.

2. As confirmed through correspondence with BPA, the portable power system monitor (PPSM) used at that time recorded frequency in intervals of 0.025 s. Probably, the researcher who extracted the digitized version from the scan had the same information about the PPSM's sampling step because a step of 0.024 152 52 s was originally reported for the digitized version.[50,51] In combination with the erroneously assumed Western reading direction of the time axis, this led to the incorrect interpretation that the time series corresponded to roughly 10 min of the pre-outage region.

However, for the reasons, stated in section S11, we assume that the time series in Figure S10 (a) does not follow the red time axis orientation, but the green one. Furthermore, we know that the record covers almost 20 h instead of roughly 10 min, but cannot determine with certainty the absolute start time of the record by comparing the digitized version and the scale paper scan. For this reason, we need to reconstruct the sampling step as well as the absolute times of the record to allow for the comparison to the real timeline of events (cf. Results). To this end, we could generate a time series from the scan based on the updated time scale information, e.g., with a software package like *DigitSeis*[52]. We do not follow this approach, but perform a comparison between the small pre-outage segment of the time series in question, i.e., the black-hatched segment in Figure S10 (a), and two pre-outage time series provided by BPA with approved absolute times. To this end, we invert the time series segment of interest, use an arbitrary time axis for this segment at the beginning, and fix a single prominent point of coincidence between both, the unknown time series from the scan and the known time series provided by BPA. In Figure S10 (b), the time scale, reconstructed based on the densely sampled time series of the original articles[50,51], is shown. Note that we use a thinned version with $N_{\text{thin}} = 11300$ data points instead of the original one, containing $N_{\text{dense}} = 23393$ samples, to cancel artificial outliers in the subsequent analyses, presented in the Results section. The data was extracted during previous unpublished works of Kamps and Ehebrecht[49] from one of the low-quality scans without readable time axis.

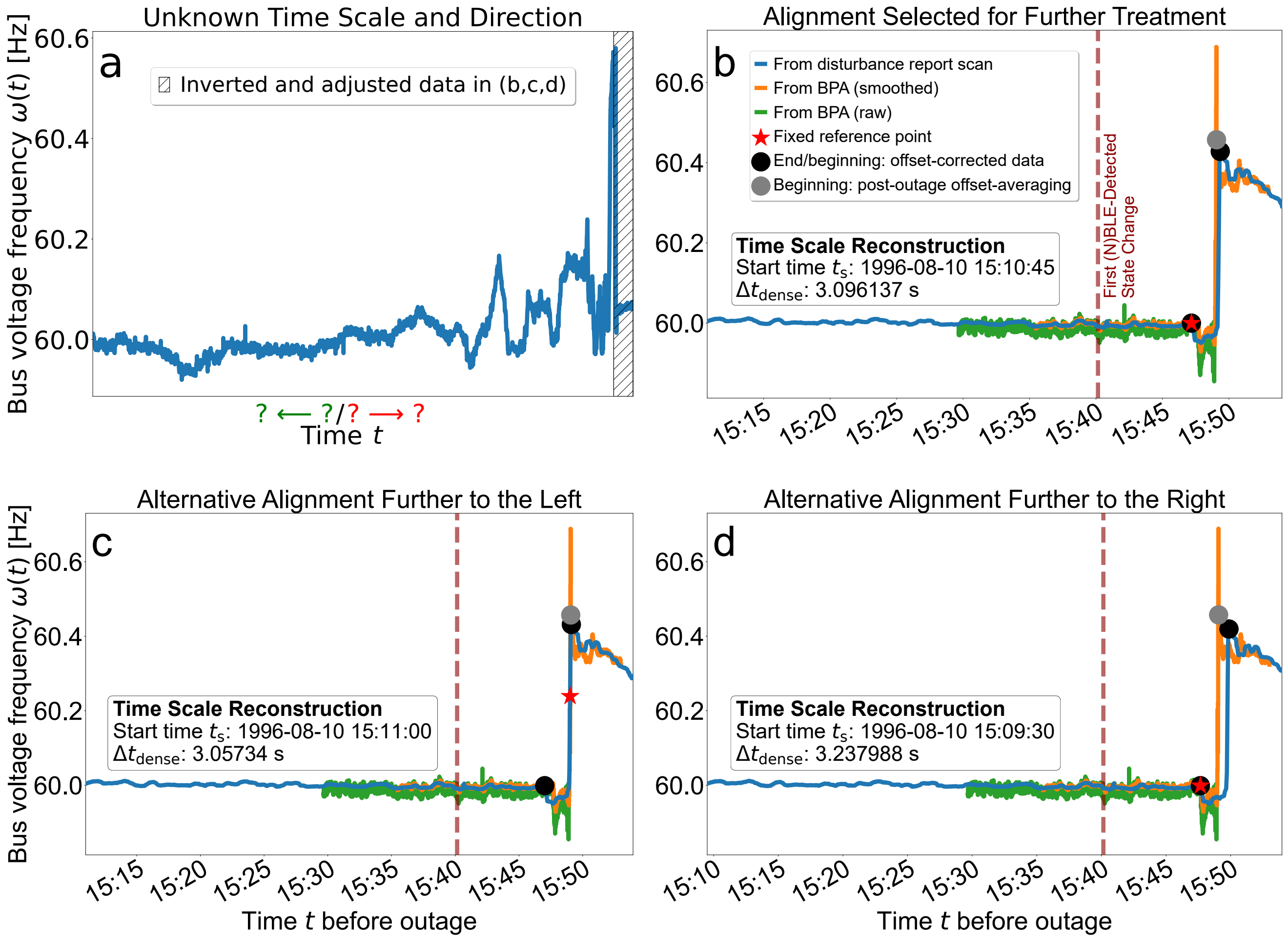

Figure S10. Reconstruction of the unknown time axis of the post-outage time series, analysed in the Results section, Figures 1(d–f). **(a)** Originally, the blue time series was reported to cover roughly 10 min in the pre-outage time interval of the NAWI cascading failure on 10th August 1996, from left to right, with the time step $\Delta t = 0.02415252\,\mathrm{s}$. An extensive search for the data source (cf. SI S11) raises doubts about the integrity of the originally assumed axis and suggests an inverted time direction, i.e., the green-marked x-axis direction instead of the red one. Furthermore, the recovered data source indicates that only the small black-hatched interval corresponds to the pre-outage period. **(b)** Therefore, we use the inverted data from the black-hatched region to align it with approved pre-outage time series that have known time scales and absolute times from BPA (green and orange). Initially, both the known and unknown time series are aligned using a prominent point marked by the red star, with an arbitrary time axis assigned to the blue time series in question. After fixing the alignment, the time step of the blue time series is adjusted to best match the approved pre-outage data from BPA. The starting time of the record and the extracted sampling step are displayed in the text box. Note that the data prior to the 59.9 Hz to 59.95 Hz dip, as well as the data of the 60.4 Hz peak, exhibit artificial offsets compared to the original source, likely resulting from the digitization process of the time series from a scan. Against this background, we correct the affected pre-outage interval, from the beginning of the time series to the first black point, by subtracting an average offset referred to 60 Hz. Similarly, we take the average of the peak interval data compared to 60.4 Hz, starting with the grey dot. The data following the second black dot are corrected by subtracting the average offset in the peak interval. The shift between the grey and black dots approximately account for time scaling discrepancies in the peaking region due to the data extraction process. **(c,d)** Alternative alignments achieved analogously to **(b)** demonstrate that the procedure introduces uncertainties in the time step on the order $\mathscr{O}(10^{-1}\mathrm{s})$ and suggests a deviation of the starting time of less than one minute. Combined with the results from SI S13, this indicates that the maximum uncertainty of the starting time is $\sigma_{t_s} = 3\,\mathrm{min}$. Thus, the alignment chosen has no influence on the results presented in the Results section.

For more details about the outlier identification and treatment, see Section S18. Nevertheless, we perform the alignment to reconstruct the time scale with the densely sampled original version. For this reason, we must convert the reconstructed time step $\Delta t_{\mathrm{dense}}$ into the thinned time step Δt_{thin} through

$$\Delta t_{\mathrm{thin}} = \frac{N_{\mathrm{dense}}}{N_{\mathrm{thin}}} \cdot \Delta t_{\mathrm{dense}} = 2.07 \cdot \Delta t_{\mathrm{dense}} \tag{S.16}$$

to get the correct time step of the BLE analysis, presented in the Results section. We fix the points exactly before the reported 59.9 Hz to 59.95 Hz frequency dip, as indicated by the red star. Because the extracted data display an offset from 60 Hz prior to the frequency dip—something not present in the original time series scan—we calculate the average data offset from 60 Hz for all data points before the first black dot and subtract it from the raw digitized version. A similar, artificial offset referred to 60.4 Hz is found in the peak region. Therefore, we average the offsets for all points beginning with the grey dot and subtract this result from the data segments starting with the second black dot. The small shift between the grey dot and the second black dot is necessary because the digitized data slightly deviate from the printed ones in the peaking region. However, the assumed shift is only an approximation.
In a final step, we stretch and compress the arbitrary time scale of the blue time series in question by keeping it fixed at the red star position. In this way, we can try various alignments between the approved time series and the blue time series, which was digitized from the report scan. The subjectively defined best fit is given by the alignment in Figure S10(b), which is subsequently prepared for the analyses in the Results section. The alignment corresponds to the time series in question,

- starting at 15:10:45 on 10th August 1996,
- with a time step of $\Delta t_{\mathrm{dense}} = 3.096\,137\,\mathrm{s}$, i.e. $\Delta t_{\mathrm{thin}} = 6.41\,\mathrm{s}$.

Two alternative alignments are shown in Figure S10(c,d) to demonstrate the uncertainties in this approach. However, the variations in the time step are at most of order $\mathcal{O}(10^{-1}\mathrm{s})$. The start times 15:11:00 and 15:09:30 in Figures S10(c,d), respectively, combined with the results from Section S13, suggest that the uncertainty in the start time is roughly $\sigma_{t_s} = \pm 3\,\mathrm{min}$. Therefore, these variations should not significantly influence our results within the order of hours. In addition to providing a suitable time axis for the actual analysis of the data, this approach serves as further support for the assumed time scale and direction, which clearly differs from those in previous studies[44,49,50,53].

S13 Start Time Uncertainty Estimate of the Post-Outage Frequency Time Series

The comparison of the outlier regions in Figure S11 reveals that the reconstructed time scale, derived in SI S12, includes an uncertainty of $\sigma_{t_s} = 3\,\mathrm{min}$. It is determined by adjusting the scale ratio of the digitized frequency time series $\omega_{\mathrm{d}}(t)$ to the screenshots of the original analogue time series $\omega_{\mathrm{sc}}(t)$ from the scan, facilitating easier comparison. The misalignment is highlighted by comparing prominent patterns in the time series, as indicated by the red arrows. The discrepancy between two of these patterns is measured between the green lines, resulting in the maximum misalignment $\sigma_{t_s} = 3\,\mathrm{min}$. An alternative approach to determine this uncertainty is shown in Figure S11(b). In this method, both time series are stacked one above the other, with the digitized version shifted to the right to match up with the time scale of the report scan. This shift is measured by the protruding end of the time series and yields the same results. Since the analysis in the Results section operates on a scale of hours, this uncertainty does not affect the conclusions in any way.

S14 Synthetic Examples Without Time Lag

The findings based on the synthetic data presented in the Results section are shown again in Figure S12. In contrast to Figure 2, the estimates are assigned to the midpoints of the windows instead of the last window points. This shifts the focus away from the online interpretation toward a technically unperturbed comparison between the estimates and the ground truth.

S15 Model Choice for the Historic NAWI Cascading Failure on 10th/11th August 1996

In Figure S13, the BLE and NBLE are compared. In particular, the noise levels, including the significant state change in the beginning of the first red interval and before the tripping of the Keeler-Allston line (end of first red interval), are nearly identical for both time series in terms of trend and magnitude, regardless of whether the BLE or the NBLE is employed. Nevertheless, there are differences in the drift slope estimates. The most noticeable difference is the quantitative shift of the drift slopes $\hat{\zeta}$ in the restoration interval. While the qualitative stabilizing trend is maintained, the values are roughly five times larger than the NBLE counterparts in the final stable plateau region, i.e., closer to zero. To better resolve the BLE's qualitatively similar trend, the drift slopes $\hat{\zeta}_{\mathrm{NBLE}}$ during the restoration interval are not shown again.

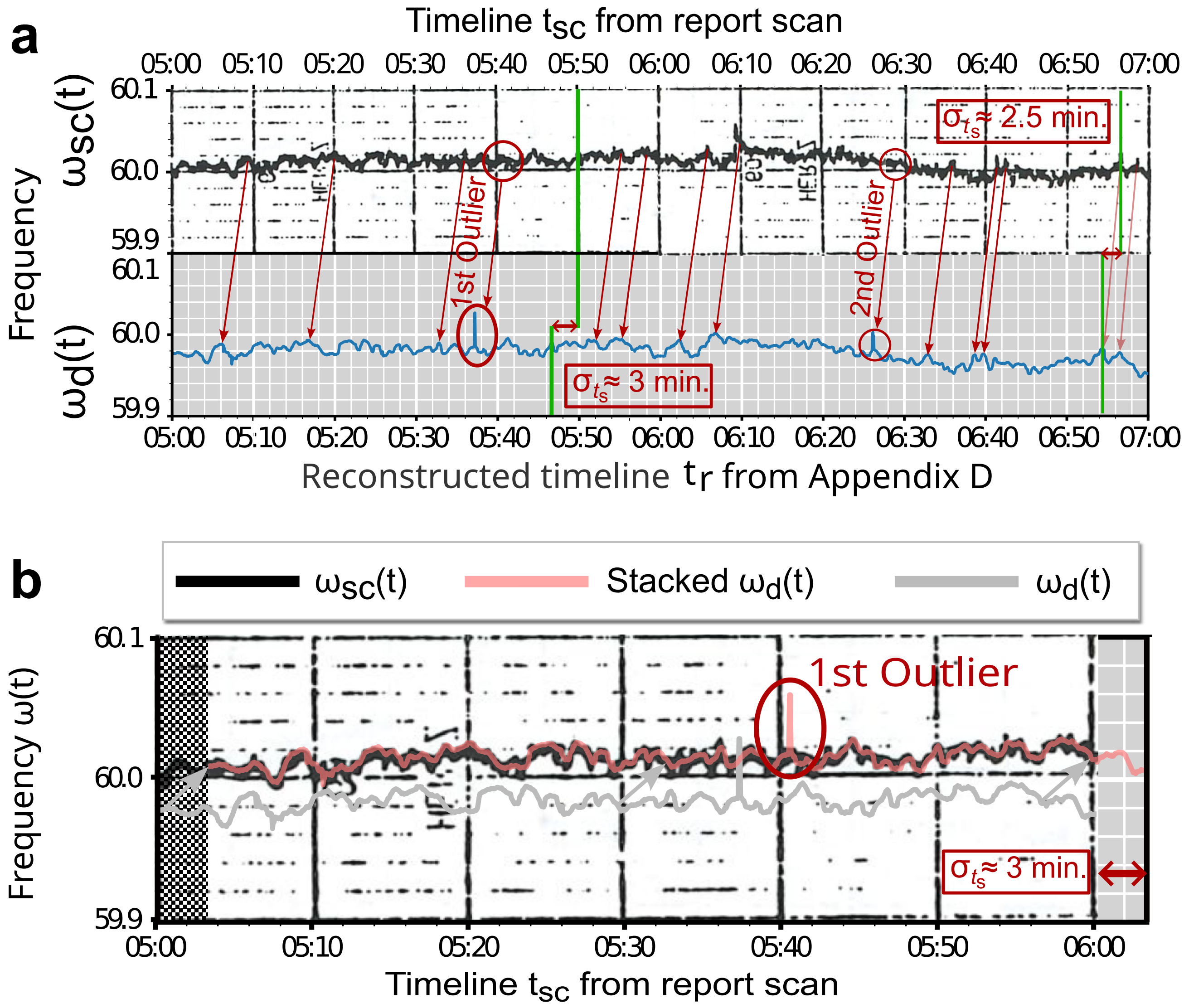

Figure S11. Identification of outlier regions and estimation of the uncertainty associated with the start time estimate of the post-outage frequency time series (cf. SI S12). The densely sampled time series is used for the comparison, but all conclusions stated here also apply to the thinned version. The scale ratio of the digitized time series is adjusted to the analogue one for an easy comparison. **(a)** Comparison between the printed original frequency time series $\omega_{sc}(t)$ and its digitized counterpart $\omega_d(t)$. The outlier regions are marked by red circles and ellipses. The prominent peaks in the digitized version are artefacts due to misinterpreting the scale paper's major grid lines as data points during extraction using a software. The two highlighted peaks are addressed using the procedure described in the Methods section. Smaller anomalies without significant impact remain, such as the two sharp dips directly behind the first red arrow beginning from the left. Key points for comparing both time series are indicated by the red arrows, which highlight discrepancies between the reconstructed time scale and the time step derived by the procedure described in SI S12. We measure the resulting uncertainty σ_{t_s} of the reconstructed start time t_s by determining the time interval between two of the prominent points in both time series, marked by the green lines. The maximum uncertainty is $\sigma_{t_s} = 3\,\text{min}$. Note that the absolute frequency values of the digitized version exhibit a negative offset of approximately $0.02\,\text{Hz}$ in the considered interval. **(b)** Additionally, we compare the first outlier region by stacking both time series segments one above the other. Note that the frequency scaling of the digitized version $\omega_d(t)$ is lost that way. However, it is another way to estimate the start time uncertainty t_s. The digitized red version is shifted to the right to match the time scale of the report, as indicated by the black-tiled area at the beginning (without digitized data) and the overhang at the end. The overhang is used to determine the uncertainty again, which aligns with the results of the previous approaches. The minute-scale uncertainty is irrelevant to the conclusions, which are drawn in the Results section, as they operate on a scale of hours.

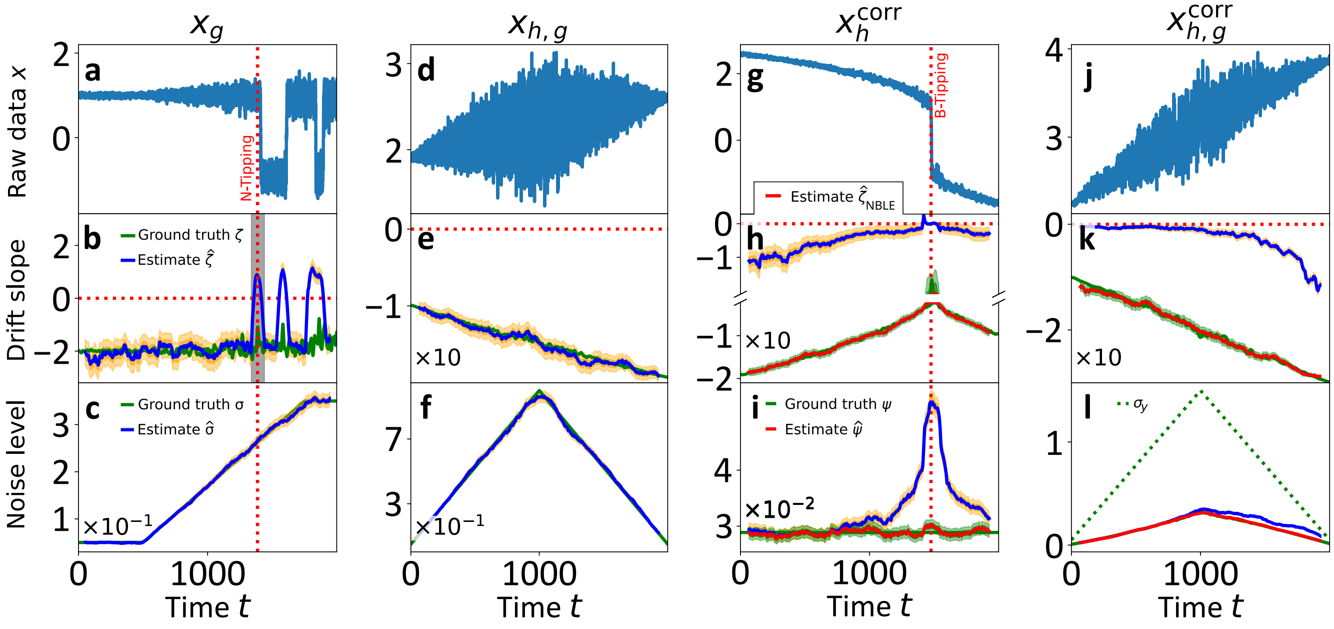

Figure S12. (N)BLE results of the synthetic data examples from the Results section of the main article, but instead of assigning the estimates of the method with the end point (cf. main article, Figure 2), the estimates are assigned to the midpoint of each window. This confirms the statement that the estimates in the Markov examples match the real values quite accurately, as shown in Figures S12(a,d). Expanding the BLE to the NBLE (red lines with green CBs) in the correlated examples (h,k) by a hidden slow OU process cancels out the significant bias of the BLE compared to the ground truths (green solid lines).

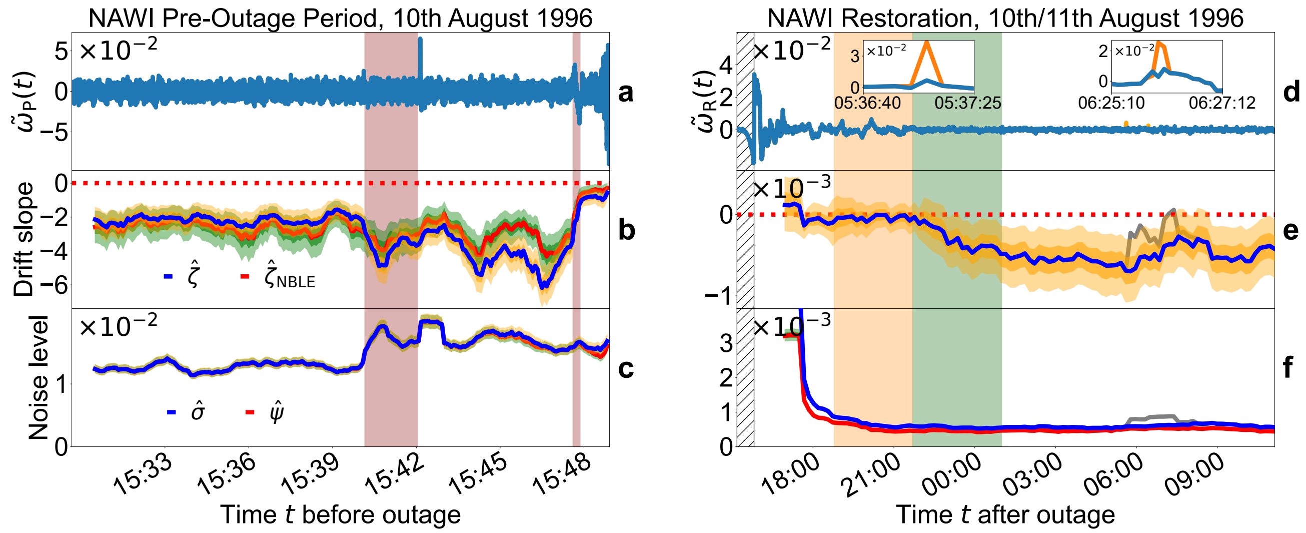

Figure S13. Comparison of the BLE and NBLE results for the bus voltage frequency data of the NAWI blackout. The BLE results closely mirror those of the NBLE. In particular, omitting the hidden OU process of the NBLE—representing potentially correlated disturbances—has minimal effect on the noise level estimates. Instead, it leads to a shift in the drift slope magnitude during the restoration interval, displaying a trend of stabilization almost identical to that of the NBLE shown in Figure 1 (not shown again to resolve the qualitatively robust trend of the BLE estimates). Furthermore, the BLE indicates a negative trend in the drift slope estimates of the pre-outage interval. Further analyses suggest that this trend is not attributable to real-world grid changes, such as control actions, but rather stems from an increasing misfit between the BLE model and the pre-outage frequency time series. For more details, please refer to the main text and Figure S14.

The seemingly oscillatory behaviour of the drift slopes after tripping the Keeler-Allston line is present in both the BLE and the NBLE. However, the drift slopes $\hat{\zeta}$ exhibit a distinctly negative trend in this oscillatory region, which is not found for the NBLE counterparts. In summary, assuming a correlated red noise process (i.e., using NBLE) does not affect the estimated noise levels, but it does impact the drift slope estimates.

Additional computations are required to answer the question which of the two models is preferable for this analysis. Therefore, approximate time intervals are defined in which the estimated drift and diffusion dynamics for the BLE and NBLE are nearly stationary. The intervals are numbered from one to three and are represented as orange data slices in the frequency graphs of Figure S14. Segments one, two, and three contain $N_{\text{seg}} = \{12000, 7280, 7399\}$ data points, respectively. Since each data slice is assumed to be explained by an individual data-generating process, the parameters (cf. Table S4) of the (N)BLE models are estimated using MCMC sampling. The obtained parameterisations are integrated to create $1.5\cdot 10^7$ samples with sampling steps Δt comparable to the originals. These samples are used to compare key statistics of the real frequency time series with those of the fitted models. Specifically, the autocorrelation functions AC(τ), the data PDFs $p(\tilde{\omega})$, and the increment PDFs $p(\tilde{\omega}_{n+1} - \tilde{\omega}_n)$ are compared. The PDFs represent Gaussian kernel estimates with a bandwidth obtained using Silverman's rule. These are evaluated with a resolution of $\Delta\omega = 8\cdot 10^{-6}$. The Python function *scipy.stats.gaussian_kde(...)*[54] is used for their derivation.

The pre-outage frequencies exhibit larger-scale oscillations (period length $T_{\tilde{\omega}_{\text{P}}} \sim 2.5\,\text{s}$) around the stable operating frequency of 60 Hz. Before the tripping of the Keeler-Allston line, a longer oscillation with a period $T'_{\tilde{\omega}_{\text{P}}} \sim 20\,\text{s}$ to 25 s may also be present. These periodic components of the signal are indicated by the AC(τ), which oscillates around zero. In particular, half of the roughly estimated period length $T_{\tilde{\omega}_{\text{P}}}$ corresponds to 25τ, where we find approximately the minimum amplitude of the AC(τ). The period length $T_{\tilde{\omega}_{\text{P}}} \sim 2.5\,\text{s}$ (i.e., 0.4 Hz) is in good agreement with known interarea oscillations of the NAWI, with a frequency around 0.2 Hz to 0.3 Hz[38].

Both the BLE and NBLE are not designed to capture such periodic features in the data. Nevertheless, if a reliable model of these frequency oscillations in the NAWI were available, it would be relatively straightforward to adapt the (N)BLE drift and diffusion functions accordingly to enhance the overall model estimation results. In some cases, it is also possible to deseasonalize the data, as shown in Ref.[8]. However, subtracting an estimate of the periodicity from the pre-outage frequency time series is not trivial, as it might also include parts of the underlying trend. These trend dynamics are encoded in the initial part of the AC(τ). We find notably correlations that tend to decrease almost linearly with increasing time lag τ. The AC(τ) with $\tau \in \{1,2,3\}$ and $\tau \in \{1,2\}$ are precisely estimated by the (N)BLE models for segment one and two, respectively.

Since the models cannot account for the periodic characteristics, they consequently tend to zero when the first AC(τ) zero crossing—i.e., the periodicity region—is reached. Therefore, we quantify the deviation of the (N)BLE-estimated AC(τ) functions from the blue raw data AC function by the sum of squared errors[55] (SSE). The SSE calculation is constrained to the area before the first zero crossing. Points included in the SSE lie in the white tile of Figure S14 and are cut for $\tau > \tau_0$ with AC(τ_0)=0 ± 0.03 to account for the approximate zero crossing of the discretized AC functions. Whereas the SSE and explanative value of the trend AC region are almost identical for the first frequency segment in both the BLE and NBLE models, the NBLE better captures the increased trend correlations in the second data segment. Although the correlation length of its hidden OU process changes only slightly from segment one to segment two (cf. Table S4), the NBLE fits better.

An estimation using the BLE would be almost identical to the OUE (cf. Section S9), which only accounts for the decrease of AC(1) from the first to the second data slice. Consequently, the decreasing $\hat{\zeta}$ can be explained using Equation S.8 and the decline in AC(1), which is equivalent to the AR1 coefficient ρ_1. This explains the gradual decrease in the BLE and OUE drift slopes $\hat{\zeta}$ from pre-outage segment one to segment two (cf. Figure S13), which is attributed to an increasing misfit between the BLE and OUE model results and the original AC function rather than to automatic stabilizing control actions or other real-world phenomena.

It remains an open question to what extent the oscillations of the second data segment—present in the BLE and NBLE drift slope estimates—are due to changing real-world dynamics or due to the limited accuracy of the models and numerics. Nevertheless, both models suggest that, aside from a noise level increase, the drift slopes exhibit greater oscillatory variation than before. This is why these oscillations should be interpreted with caution; however, they are presumed to be related to deterministic changes that result in stronger variations in stability levels in the second segment compared to the first.

Furthermore, the approximately linear behaviour of the AC at the beginning (reflecting a considerable correlation length), the downshift of AC($\tau = 1$), the notable uplift of AC(τ) for $\tau \geq 3$, and the intrinsic periodicity are signs of complex processes that are resolved by the very fine sampling with $\Delta t = 0.05\,\text{s}$. Notably, complex phenomena of the frequency dynamics, emerging on subsecond time scales, are already observed by Schäfer et al.[14]. The authors extract frequency power laws $f^{\tilde{\beta}}$ from frequency data and find exponents $\tilde{\beta}_{>1\,\text{s}} \sim -2.5$ for coarse scales above one second, and a flattening or even increasing power spectra for subsecond scales, with $-2 \lesssim \tilde{\beta}_{<1\,\text{s}} \lesssim 2$. An analysis, analogue to Ref.[14] in the same frequency range, yields exponents with $\tilde{\beta}_{>1\,\text{s}} \approx -1.27$, $\tilde{\beta}_{<1\,\text{s}} \approx -0.79$ ($\tilde{\beta}_{>1\,\text{s}} \approx -1.32$, $\tilde{\beta}_{<1\,\text{s}} \approx -0.79$ without detrending) for the first segment, and $\tilde{\beta}_{>1\,\text{s}} \approx -1.20$, $\tilde{\beta}_{<1\,\text{s}} \approx -0.75$ ($\tilde{\beta}_{>1\,\text{s}} \approx -1.27$, $\tilde{\beta}_{<1\,\text{s}} \approx -0.72$) for the second one. For the entire time series, the exponents are $\tilde{\beta}_{>1\,\text{s}} \approx -1.69$, $\tilde{\beta}_{<1\,\text{s}} \approx -0.76$ ($\tilde{\beta}_{>1\,\text{s}} \approx -1.84$, $\tilde{\beta}_{<1\,\text{s}} \approx -0.76$). Although these extracted exponents differ quantitatively from those found for

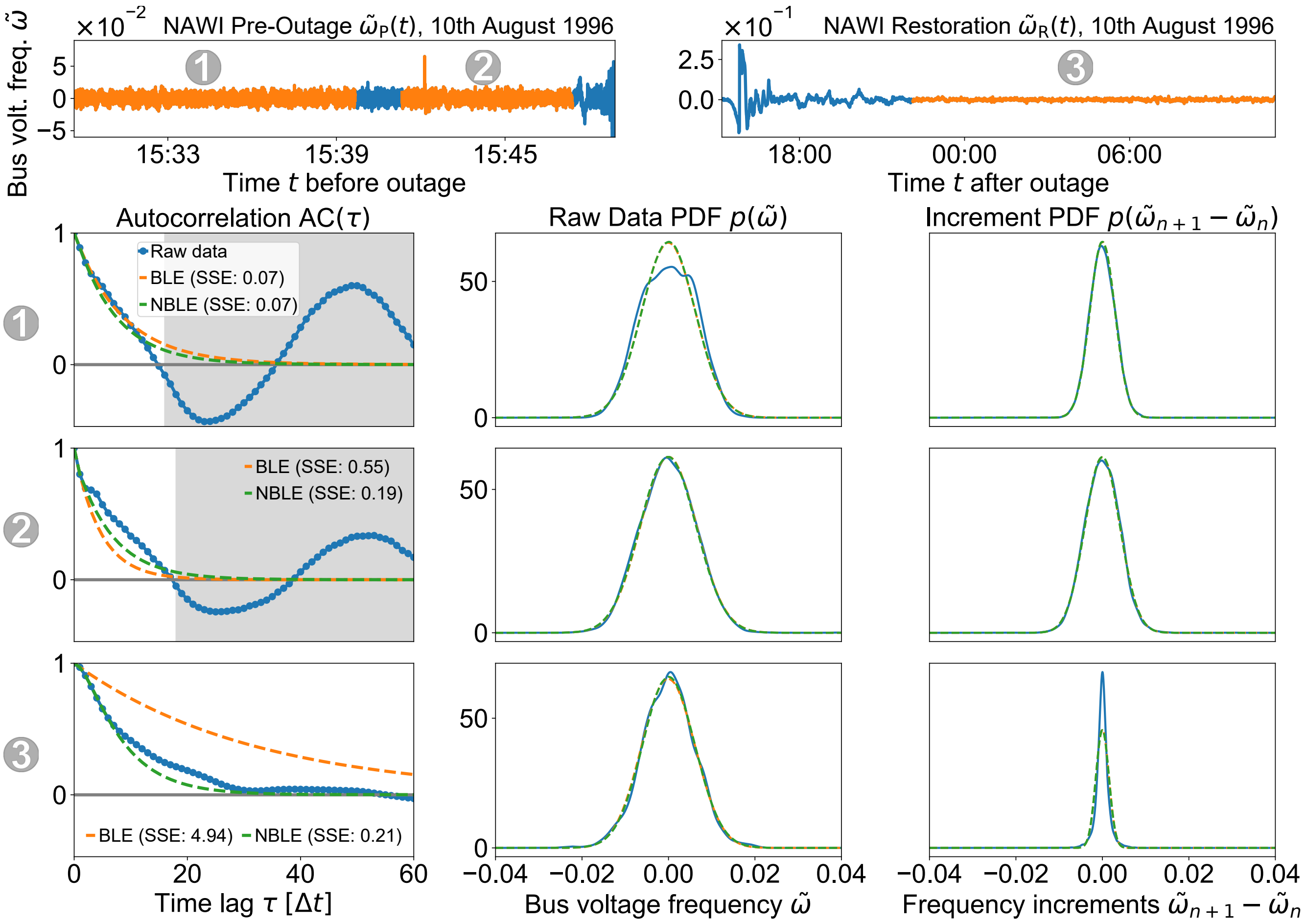

Figure S14. Comparison of the autocorrelation AC(τ), data PDFs, and increment PDFs across three segments (orange and numbered from one to three) of the NAWI blackout frequency time series, which are assumed to be stationary and characterized by different power grid states. The deviation of the AC(τ) estimates from the data before reaching the periodicity region (grey-shaded area) is quantified as SSE. The increasing misfit between the original AC(τ) and that of the BLE model from the first to the second pre-outage frequency segment results in a shift in the BLE drift slope estimates (cf. Figure S13). The hidden fast-scale OU process in the NBLE better fits the second data slice. Notably, the NBLE model significantly improves the match with the AC(τ) of the restoration interval by incorporating a strongly correlated hidden OU process (cf. Table S4). The data and increment PDFs for all segments, represented as kernel density estimates, are reasonably well captured by both the BLE and NBLE models. The increment PDFs in the pre-outage interval are particularly precise, while those in the restoration period show slightly more deviation, but remain adequate. In summary, the NBLE is favoured based on this comparison.

Data Segment	Estimation Scheme	Model Parameters					
		θ_0	θ_1	θ_2	θ_3	θ_4	θ_5
**1**	BLE	$-8.72562 \cdot 10^{-5}$	-2.21257	2.70063	$-5.07130 \cdot 10^{-1}$	$1.26961 \cdot 10^{-2}$	—
	NBLE	$3.73489 \cdot 10^{-5}$	-2.60544	$5.76980 \cdot 10^{-2}$	$-2.53568 \cdot 10^{-2}$	$5.91165 \cdot 10^{-2}$	$2.33541 \cdot 10^{-1}$
**2**	BLE	$-2.65156 \cdot 10^{-5}$	-3.94076	2.00736	$8.01646 \cdot 10^{-1}$	$1.73015 \cdot 10^{-2}$	—
	NBLE	$-3.23303 \cdot 10^{-4}$	-2.87212	2.24154	1.32572	$7.07301 \cdot 10^{-2}$	$2.06745 \cdot 10^{-1}$
**3**	BLE	$-2.37119 \cdot 10^{-7}$	$-4.67147 \cdot 10^{-3}$	$3.41127 \cdot 10^{-3}$	-1.15331	$5.88723 \cdot 10^{-4}$	—
	NBLE	$9.15296 \cdot 10^{-7}$	$-2.23028 \cdot 10^{-2}$	$1.32595 \cdot 10^{-2}$	3.69809	$3.28621 \cdot 10^{-4}$	4.32873

Table S4. Estimated parameters rounded to six significant digits for the BLE and the NBLE applied to the three orange data segments indicated in Figure S14. The parameters correspond to the mode of the parameter PDFs. The squared parameter θ_5^2 is an estimate of the correlation length of the NBLE's hidden OU process.

other grids[14], they similarly exhibit a flattening behaviour for the subsecond scale. Furthermore, the spectra reflect periodic features, showing peaks roughly around 0.2 Hz and 0.4 Hz. The latter coincides with the previously mentioned period lengths and is essentially the second harmonic of the former.

Considering the AC functions of the frequencies during the restoration interval confirms that the NBLE is significantly better suited for these data. Noteworthy, there is an absence of periodic contributions in the data, likely related to the much coarser resolution: The restoration interval is sampled with a time step $\Delta t \approx 6.41\,\mathrm{s}$ (cf. Section S12), in contrast to $\Delta t = 0.05\,\mathrm{s}$ in the pre-outage period. Moreover, the NBLE model involves a notable correlation in the hidden OU process during the restoration interval (cf. Table S4). The data and increment PDFs for the BLE and NBLE are almost identical. In the pre-outage period, especially the first differences are well-fitted. However, in the second data segment, the NBLE PDFs match the mode slightly better. During the restoration interval, the estimated PDFs deviate generally more from the raw data PDFs.

The findings were obtained by comparing models fitted on data segments that contain significantly more data points than the rolling windows used in the analysis. To assess robustness, we also examined several randomly selected rolling windows of size $N_w = 1000$ data points. While the results remain robust, there was slightly more variation from window to window. The AC variations tend to increase with higher time lags due to the progressively thinner dataset, eventually rendering statistical computations unfeasible.

S16 Robustness to Window Size Variation

In this section, we analyse the sensitivity of the (N)BLE results to window size variation for the NAWI outage time series and the simulated NAWI models, presented in Figures 1 and S3, respectively. The results are shown in Figure S15.

The window sizes in Figure S15(a–h) are varied over a wide range of $\pm 50\,\%$ based on the window sizes used in the main analyses, i.e., 1000 and 8000 samples for the real-world and synthetic data, respectively. For the modelled McNary loss, however, we restrict the window size enlargement to $12.5\,\%$. This restriction ensures that the drift slope estimates are not perturbed by either the peak caused by the McNary loss or the primary control action at the beginning and end of the red-shaded interval. Even slightly larger windows, e.g., with 10^4 points, would always include one or both of these disturbances.

The analysis reveals that the characteristic fingerprints of the NAWI pre-outage data starting at the red-shaded intervals shown in Figure S15(a,b), as well as their time stamps, are largely independent of window size. In particular, the time at which the noise amplitude increases to a permanently elevated level (cf. dimension line with double-headed arrows in Figure 1(d)) remains unchanged regardless of the chosen window size. Similarly, in Figure S15(c,d), the restoration process of the NAWI is consistently reproduced across all considered time window sizes. Moreover, the behaviour of the (N)BLE estimates for the NAWI models, presented in Figure S15(e–j), remains robust to variations in window size.

Furthermore, the differing behaviour of the BLE and NBLE drift slopes in the Keeler-Allston line THIF scenario (cf. Figure S15(g)) is a persistent feature, as is the decreased local stability following the loss of the Mcnary power units in Figure S15(i). The beginning of the red-shaded interval in Figure S15(i) marks the loss of the McNary units, which causes a peak in the data. Initially, this peak results in an estimation artefact, visible as a sudden jump to a plateau of one window length (cf. short blue to long black plateau). However, *after* this plateau, subsequent estimates—computed from unperturbed windows within the red-shaded interval—remain elevated, indicating a less stable power grid state.

Finally, typical characteristics of rolling window methods are observed throughout the results, including an increased time lag in manifesting trends and a dampened impact of outliers for larger window sizes.

S17 Frequency Dip and Changing Grid State

The time stamp of the first pre-outage change of the power grid state, as indicated by the BLE in Figure 1(b,c), is marked as a dark-red dashed vertical line in Figure S10(b–d). The time stamp coincides with the downward trend of a frequency dip, best visible in the green and orange pre-outage time series provided by BPA. The frequency dip might indicate sudden load increase that might be triggered by the beginning of the Keeler-Allston line's THIF or by another reason. Even though the actual reason cannot be deduced with certainty, the comparison suggests a relationship between the frequency dip and the permanent change in the grid state identified by the (N)BLE, occurring roughly 2 min before the officially declared triggering event of the historic NAWI cascading failure on 10th August 1996.

S18 Identification of Artificial Outliers in the Post-Outage Frequency

We noticed strange artefacts in the (N)BLE results, namely discontinuous jumps of the slope $\hat{\zeta}_{(\mathrm{NBLE})}$ and noise estimates $\hat{\sigma}$ $(\hat{\Psi})$ to values that persist over almost one window length, before returning to their previous values. Based on this observation, we inspected the affected time series periods carefully by eye. Therefore, we zoomed in on the affected time periods and indeed identified significant outlier shocks. Thanks to the approved report's frequency time series scan[17] $\omega_{\mathrm{sc}}(t)$ from Exhibit 10, we

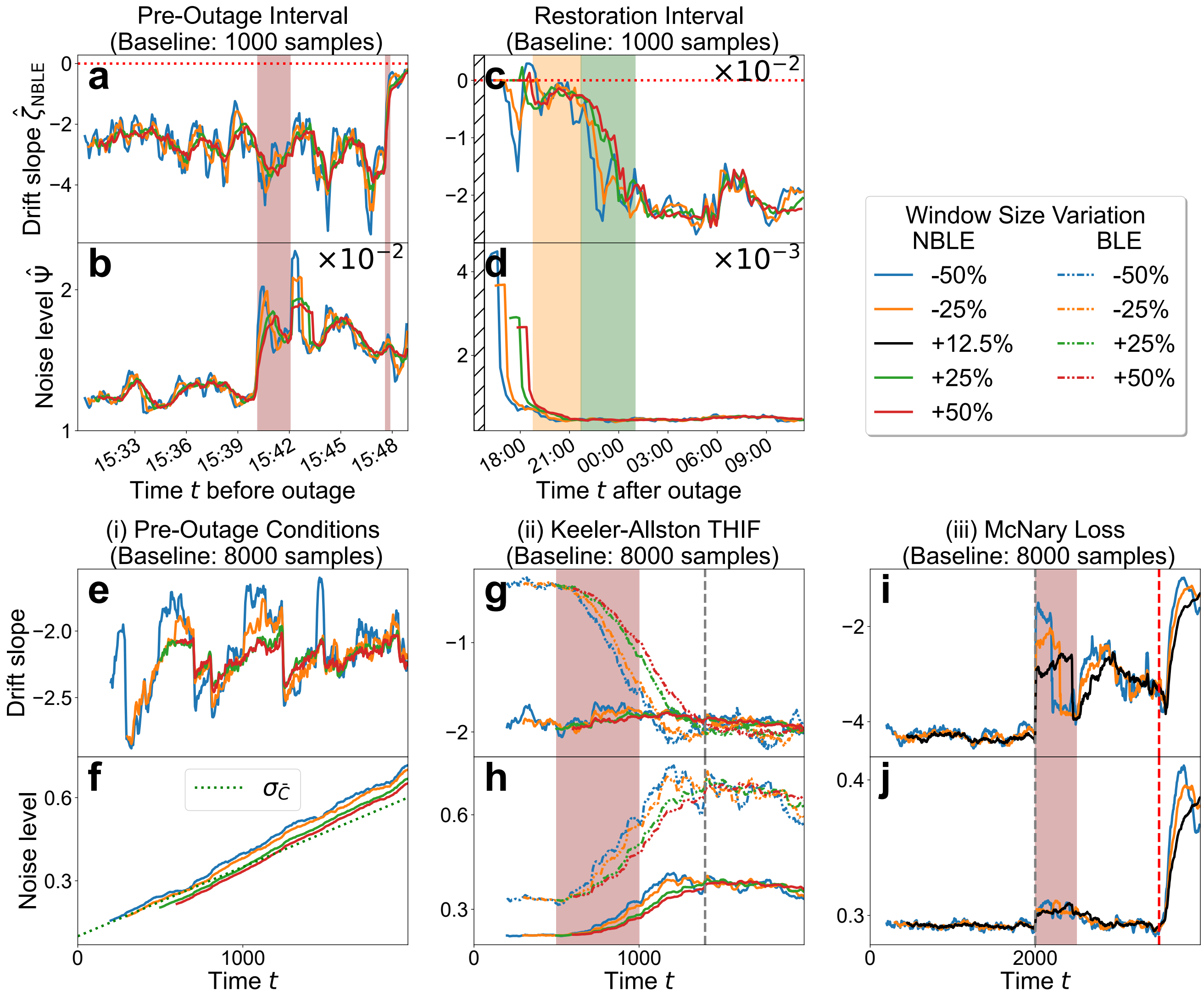

Figure S15. Window size sensitivity analysis for **(a,b)** the NAWI pre-outage interval, **(c,d)** the NAWI restoration interval, and for the simulated NAWI outage episodes: **(e,f)** the pre-outage conditions, **(g,h)** the Keeler-Allston line THIF, and **(i,j)** the loss of the McNary power units. Shaded time intervals and vertical orientation lines are defined as in Figures 1 and S3. The baseline window sizes used in the analyses are symmetrically varied by $\pm 50\,\%$ in all cases, except for the McNary loss scenario (**(i,j)**), where the maximum increase is limited to $+12.5\,\%$. This restriction ensures that the computations include rolling windows unaffected by data peaks caused by the McNary unit loss or by primary control actions at the beginning and end of the red-shaded time interval. Apart from typical characteristics of rolling window methods—such as slower trend manifestation and dampened impact of outliers for larger windows—the (N)BLE results are almost invariant to window size. In particular, the time stamps of crucial changes in drift slopes and noise levels at the beginning of the red-shaded interval in **(a,b)** are entirely unaffected by window size. Similarly, the continuous re-stabilization and noise level decrease over the green- and orange-shaded intervals in **(c,d)**, respectively, show little to no dependence on window size. In **(e–j)**, the characteristic (N)BLE fingerprints observed in the NAWI model simulations (cf. SI S3 and Figure S3) persist across the considered window size variations. Importantly, in **(i,j)**, the drift slopes consistently increase at the end of the red-shaded interval, once the estimation artefact—lasting one window length and originating at the start of the red-shaded area—is excluded.

were able to compare the original printed version $\omega_{\mathrm{sc}}(t)$ to the digitized version $\omega_{\mathrm{d}}(t)$.

In Figure S11, a comparison of the time segment from 05:00 to 07:00 on 11th August 1996 is provided. For easier comparison, we adjusted the scale ratio of the digitized frequency time series $\omega_{\mathrm{d}}(t)$ to that given by the snapshots of the printed frequency scan $\omega_{\mathrm{sc}}(t)$. Note that there is a time series shift in Figure S11(a) due to the approximate reconstruction of the start time of the post-outage frequency data (cf. Section S12). More details on the start time uncertainty and the observed shift of approximately

3 min can be found in Section S13.

Taking this shift into account, we can now identify the corresponding regions in both the originally printed and the digitized frequency data, $\omega_{sc}(t)$ and $\omega_d(t)$, respectively, marked by the red circles and ellipses in Figure S11(a). The first outlier is additionally highlighted in Figure S11(b), which provides a more straightforward method of comparison by stacking both equally scaled time series one above the other. However, the results in Figure S11(a) emphasize that the total values of the frequencies are only approximately reconstructed. Specifically, the digitized frequencies $\omega_d(t)$ are negatively shifted by an offset of roughly 0.02 Hz.

In summary, the outliers are generated by the digitizing software, which tends to misinterpret the scale paper grid, particularly the major lines, as data points. The denser the chosen sampling, the more pronounced this effect becomes. For this reason, we utilize a digitized version with only 11300 data points instead of the original version with 23393 data points used in previous articles[44,49–51]. By doing so, we effectively eliminate four of the six prominent outliers introduced during the data generation process, although smaller outliers with less impact remain, such as the two sharp dips in the downward trend of the frequency directly behind the first red arrow from the left. These smaller outliers are most likely caused by the minor grid lines. The remaining two outliers highlighted in Figure S11 are addressed by the procedure described in the Methods section. Briefly, the outliers are replaced with random values in a suitable range.

S19 Analysis Details

The most important parameters for the (N)BLE analyses presented in the main article are summarized in Table S5. Additional information and a comprehensive collection of Python scripts to reproduce the analyses are available at `https://github.com/MartinHessler/Disentangling_Tipping_Types` in the folder “Quantifying Local Stability and Noise Levels”.

**Analysis**	**Method**	**Number of MC Samples**	**Prior Range**					
			θ_0	θ_1	θ_2	θ_3	θ_4	θ_5
**Fig. 1(b–d)**	BLE<br>NBLE	$5\cdot10^3$<br>$1.5\cdot10^4$	$[-50,50]$<br>$[-50,50]$	$[-50,50]$<br>$[-50,50]$	$[-50,50]$<br>$[-50,50]$	$[-50,50]$<br>$[-50,50]$	$[0,50]$<br>$[0,50]$	—<br>$[0,50]$
**Fig. 1(e–g)**	BLE<br>NBLE	$1.5\cdot10^4$<br>$1.5\cdot10^4$–$8\cdot10^5$	$[-50,50]$<br>$[-100,100]$	$[-50,50]$<br>$[-100,100]$	$[-50,50]$<br>$[-100,100]$	$[-50,50]$<br>$[-100,100]$	$[0,50]$<br>$[0,100]$	—<br>$[0,200]$
**Fig. 2(a–c)**	BLE	$5\cdot10^3$	$[-50,50]$	$[-50,50]$	$[-50,50]$	$[-50,50]$	$[0,50]$	—
**Fig. 2(d–f)**	BLE	10^4	$[-50,50]$	$[-50,50]$	$[-50,50]$	$[-50,50]$	$[0,50]$	—
**Fig. 2(g–i)**	BLE<br>NBLE	10^4<br>$3\cdot10^5$	$[-50,50]$<br>$[-100,100]$	$[-50,50]$<br>$[-100,100]$	$[-50,50]$<br>$[-100,100]$	$[-50,50]$<br>$[-100,100]$	$[0,50]$<br>$[0,100]$	—<br>$[0,200]$
**Fig. 2(j–l)**	BLE<br>NBLE	$1.5\cdot10^4$<br>$2\cdot10^4$–$8\cdot10^5$	$[-50,50]$<br>$[-100,100]$	$[-50,50]$<br>$[-100,100]$	$[-50,50]$<br>$[-100,100]$	$[-50,50]$<br>$[-100,100]$	$[0,50]$<br>$[0,100]$	—<br>$[0,200]$

Table S5. MCMC parameters used for the analyses in Figures 1 and 2. Ranges in the number of samples per Markov chain indicate the minimum and maximum chain length utilized to reach convergence over all windows. All MCMC computations are performed by applying 50 walkers and omitting the first 200 Monte Carlo samples as a burn-in. The burn-in period avoids transients in the Markov chains. The prior ranges were chosen large enough to ensure that the sampled parameter distributions are not artificially truncated. The parameters θ_4 and θ_5 are restricted to the positive domain by their physical interpretation as noise amplitudes. The MCMC sampling starts from maximum a posteriori solutions obtained from the following initial guesses for the model parameters: $\underline{\theta}^{\mathrm{BLE}}_{\mathrm{init}} = (1,1,1,1,1)^{\mathrm{T}}$ for the BLE, and $\underline{\theta}^{\mathrm{NBLE}}_{\mathrm{init}} = (0,1,0,0,0,0.1)^{\mathrm{T}}$ and $\underline{\theta}^{\mathrm{NBLE}}_{\mathrm{init}} = (0,1,0,0,0.7,3)^{\mathrm{T}}$ for the NBLE in Figures 1 and 2, respectively.

SI References

1. Heßler, M. Supplementary GitHub repository for "Quantifying local stability and noise levels from time series in the US Western Interconnection blackout on 10th August 1996". https://github.com/MartinHessler/Disentangling_Tipping_Types, DOI: 10.5281/zenodo.15282709 (2025).
2. Heßler, M. antiCPy. https://github.com/MartinHessler/antiCPy, DOI: 10.5281/zenodo.6046562 (2021).
3. Heßler, M. antiCPy's documentation. https://anticpy.readthedocs.io (2021).
4. Perretti, C. T. & Munch, S. B. Regime shift indicators fail under noise levels commonly observed in ecological systems. *Ecol. Appl.* **22**, 1772–1779, DOI: 10.1890/11-0161.1 (2012).
5. Gsell, A. S. *et al.* Evaluating early-warning indicators of critical transitions in natural aquatic ecosystems. *Proc. Natl. Acad. Sci.* **113**, E8089–E8095, DOI: 10.1073/pnas.1608242113 (2016).
6. Boerlijst, M. C., Oudman, T. & de Roos, A. M. Catastrophic collapse can occur without early warning: Examples of silent catastrophes in structured ecological models. *PLoS ONE* **8**, e62033, DOI: 10.1371/journal.pone.0062033 (2013).
7. Hastings, A. & Wysham, D. B. Regime shifts in ecological systems can occur with no warning. *Ecol. Lett.* **13**, 464–472, DOI: 10.1111/j.1461-0248.2010.01439.x (2010).
8. Heßler, M. & Kamps, O. Quantifying resilience and the risk of regime shifts under strong correlated noise. *PNAS Nexus* **2**, pgac296, DOI: 10.1093/pnasnexus/pgac296 (2022). https://academic.oup.com/pnasnexus/article-pdf/2/2/pgac296/49084750/pgac296.pdf.
9. Crauel, H. & Flandoli, F. Additive noise destroys a pitchfork bifurcation. *J. Dyn. Differ. Equations* **10**, 259–274, DOI: 10.1023/a:1022665916629 (1998).
10. Haken, H. *Synergetics: Introduction and Advanced Topics*. Physics and Astronomy Online Library (Springer, 2004).
11. Kundur, P. *Power System Stability and Control* (McGraw-Hill, New York, et al., 2007), reprint edn.
12. Schäfer, B., Beck, C., Aihara, K., Witthaut, D. & Timme, M. Non-Gaussian power grid frequency fluctuations characterized by Lévy-stable laws and superstatistics. *Nat. Energy* **3**, 119–126, DOI: 10.1038/s41560-017-0058-z (2018).
13. Ulbig, A., Borsche, T. S. & Andersson, G. Impact of low rotational inertia on power system stability and operation. *IFAC Proc. Vol.* **47**, 7290–7297, DOI: 10.3182/20140824-6-za-1003.02615 (2014).
14. Schäfer, B. *et al.* Microscopic fluctuations in power-grid frequency recordings at the subsecond scale. *Complexity* **2023**, 1–13, DOI: 10.1155/2023/2657039 (2023).
15. Filatrella, G., Nielsen, A. H. & Pedersen, N. F. Analysis of a power grid using a Kuramoto-like model. *The Eur. Phys. J. B* **61**, 485–491, DOI: 10.1140/epjb/e2008-00098-8 (2008).
16. Menck, P. J., Heitzig, J., Kurths, J. & Joachim Schellnhuber, H. How dead ends undermine power grid stability. *Nat. Commun.* **5**, DOI: 10.1038/ncomms4969 (2014).
17. Watkins (BPA), D. *et al.* Western Systems Coordinating Council. Disturbance Report. For the Power System Outage that Occurred on the Western Interconnection. August 10, 1996. 15:48 PAST. Approved by the WSCC Operation Commitee on October 18, 1996 (1996). The text source without Appendices was provided by Bonneville Power Administration via a Freedom of Information Act request thanks to James King (FOIA Public Liaison, BPA; P.O. Box 3621, CGI-7, Portland, OR 97208-3621; phone: 503-230-7621; email: FOIA@bpa.gov) and Brian Roth (FOIA Case Coordinator, BPA). The Appendices 2, 3, 5, and 9 of the timeline were provided thanks to Mary Schaff from the Washington State Library, currently operating under the Secretary of State, Steve Hobbs, through mail correspondence via the "Ask a Librarian" service (Washington State Library, Point Plaza East, 6880 Capitol Blvd. SE, Tumwater, PO Box 42460, Olympia WA 98504-2460; phone: (360) 704-5200; email: askalibrarian@sos.wa.gov). A low-quality scan of the restoration frequency time series from the approved report's Exhibit 10 was provided by WECC, and a readable version from the preliminary report was made available thanks to Jeanie Fisher from the Seattle Municipal Archives (Seattle Municipal Archives, 600 Fourth Avenue, Third Floor, Seattle, WA, 98104, PO Box 94728, Seattle, WA, 98124-4728; phone: (206) 684-8353; email: archives@seattle.gov). Contact with Jeanie Fisher of the Seattle Municipal Archives was established by Mary Schaff.
18. Bahador, N., Namdari, F. & Matinfar, H. R. Tree-related high impedance fault location using phase shift measurement of high frequency magnetic field. *Int. J. Electr. Power & Energy Syst.* **100**, 531–539, DOI: 10.1016/j.ijepes.2018.03.008 (2018).
19. Bahador, N., Namdari, F. & Matinfar, H. R. Modelling and detection of live tree-related high impedance fault in distribution systems. *IET Gener. Transm. & Distribution* **12**, 756–766, DOI: 10.1049/iet-gtd.2017.0211 (2018).

20. Oberhofer, U. *et al.* Non-linear, bivariate stochastic modelling of power-grid frequency applied to islands. *arXiv* DOI: 10.1109/powertech55446.2023.10202986 (2023). 2301.04551.

21. Friedrich, R. & Peinke, J. Description of a turbulent cascade by a Fokker-Planck equation. *Phys. Rev. Lett.* **78**, 863–866, DOI: 10.1103/PhysRevLett.78.863 (1997).

22. Siegert, S., Friedrich, R. & Peinke, J. Analysis of data sets of stochastic systems. *Phys. Lett. A* **243**, 275–280, DOI: https://doi.org/10.1016/S0375-9601(98)00283-7 (1998).

23. Friedrich, R. *et al.* Extracting model equations from experimental data. *Phys. Lett. A* **271**, 217–222, DOI: 10.1016/s0375-9601(00)00334-0 (2000).

24. European Commission (adopted the regulation). COMMISSION REGULATION (EU) 2017/1485 of 2 August 2017 establishing a guideline on electricity transmission system operation (Text with EEA relevance). *Off. J. Eur. Union* (2021). https://eur-lex.europa.eu/legal-content/EN/TXT/?uri=CELEX%3A02017R1485-20210315. European Legislation Identifier (ELI): http://data.europa.eu/eli/reg/2017/1485/2021-03-15 (Retrieved: 30 April 2025).

25. 50Hertz Transmission GmbH, Amprion GmbH, TransnetBW GmbH, Tennet TSO GmbH. Prequalification Process for Balancing Service Providers (FCR, aFRR, mFRR) in Germany ("PQ conditions"). https://www.regelleistung.net/xspproxy/api/StaticFiles/Regelleistung/Infos_f%C3%BCr_Anbieter/Wie_werde_ich_Regelenergieanbieter_Pr%C3%A4qualifikation/Pr%C3%A4qualifikationsbedingungen_FCR_aFRR_mFRR/PQ-Bedingungen-03.06.2022(englisch).pdf (Retrieved: 26 May 2024) (2022).

26. Verband der Netzbetreiber – VDN – e.V. beim VDEW. TransmissionCode 2003 Anhang D 1: Unterlagen zur Präqualifikation für die Erbringung von Primärregelleistung für die ÜNB (Stand August 2003). https://www.vde.com/resource/blob/937784/46070ff6f906891b65d537ae8a648c5b/transmissioncode-2007--netz--und-systemregeln-der-deutschen-uebertragungsnetzbetreiber-anhang-d1-data.pdf (Retrieved: 30 April 2025).

27. Ippolito, M. G., Musca, R. & Zizzo, G. Analysis and simulations of the primary frequency control during a system split in continental Europe power system. *Energies* **14**, 1456, DOI: 10.3390/en14051456 (2021).

28. Gorjão, L. R., Schäfer, B., Witthaut, D. & Beck, C. Spatio-temporal complexity of power-grid frequency fluctuations. *New J. Phys.* **23**, 073016, DOI: 10.1088/1367-2630/ac08b3 (2021).

29. Gloe, A., Jauch, C. & Räther, T. Grid support with wind turbines: The case of the 2019 blackout in Flensburg. *Energies* **14**, 1697, DOI: 10.3390/en14061697 (2021).

30. Lin, Y. T., Tian, Y., Anghel, M. & Livescu, D. Data-driven learning for the Mori-Zwanzig formalism: a generalization of the Koopman learning framework. *arXiv* DOI: 10.48550/ARXIV.2101.05873 (2021). 2101.05873.

31. Darve, E., Solomon, J. & Kia, A. Computing generalized Langevin equations and generalized Fokker–Planck equations. *Proc. Natl. Acad. Sci.* **106**, 10884–10889, DOI: 10.1073/pnas.0902633106 (2009).

32. Center for Sustainable Systems, University of Michigan. U.S. Energy System Factsheet. Pub. No. CSS03-11. https://css.umich.edu/publications/factsheets/energy/us-energy-system-factsheet (Retrieved: 13 April 2023) (2021).

33. Center for Sustainable Systems, University of Michigan. U.S. Renewable Energy Factsheet. Pub. No. CSS03-12. https://css.umich.edu/publications/factsheets/energy/us-renewable-energy-factsheet (Retrieved: 13 April 2023) (2021).

34. Eakeley, E. C. *et al.* 1996 System Disturbances. Review of Selected 1996 Electric System Disturbances in North America. https://www.nerc.com/pa/rrm/ea/SystemDisturbanceReportsDL/1996SystemDisturbance.pdf (Retrieved: 13 April 2023) (2002). North American Electric Reliability Council. Princeton Forrestal Village 116-390 Village Boulevard Princeton, New Jersey 08540-5731.

35. Harrison, J. Blackout of 1996. https://www.nwcouncil.org/reports/columbia-river-history/blackout/ (Retrieved: 13 April 2023) (2023). Northwest Power and Conservation Council.

36. Venkatasubramanian, M. V. PSERC Background Paper. Analyzing Blackout Events: Experience from the Major Western Blackouts in 1996. https://documents.pserc.wisc.edu/documents/publications/special_interest_publications/grid_reliability/Venkatasubramanian_Investigation_PSERC_Aug_2003.pdf (Retrieved: 30 April 2025) (2003). Power Systems Engineering Research Center, 428 Phillips Hall, Cornell University, Ithaca, NY 14853-5401.

37. Venkatasubramanian, M. V. & Li, Y. Analysis of 1996 western American electric blackouts. In *Bulk Power System Dynamics and Control*, vol. VI of *IREP Symposium on Bulk Power System Dynamics and Control - Managing Complexity in Power Systems: From Micro-Grids to Mega-Interconnections*, 685–721, DOI: 10.1103/RevModPhys.94.015005 (Cortina d'Ampezzo, Italy, 2004).

38. Kosterev, D., Taylor, C. & Mittelstadt, W. Model validation for the August 10, 1996 WSCC system outage. *IEEE Transactions on Power Syst.* **14**, 967–979, DOI: 10.1109/59.780909 (1999).

39. Hauer, J. F. & Burns, J. W. Roadmap to Monitor Data Collected during the WSCC Breakup of August 10, 1996. https://www.pnnl.gov/main/publications/external/technical_reports/PNNL-19459.pdf (Retrieved: 13 April 2023) (2010). Richland, WA: Pacific Northwest National Laboratory.

40. WSCC Investigative Task Force. WSCC Preliminary System Disturbance Report. August 10,1996. 15:48 PAST (1996). The source was provided via mail correspondence thanks to Jeanie Fisher from the Seattle Municipal Archives (Seattle Municipal Archives, 600 Fourth Avenue, Third Floor, Seattle, WA, 98104, PO Box 94728, Seattle, WA, 98124-4728; phone: (206) 684-8353; email: archives@seattle.gov). Contact was made thanks to Mary Schaff (contacted via the "Ask a Librarian" service) from the Washington State Library, currently operating under the Secretary of State, Steve Hobbs. (Washington State Library, Point Plaza East, 6880 Capitol Blvd. SE, Tumwater, PO Box 42460, Olympia WA 98504-2460; phone: (360) 704-5200; email: askalibrarian@sos.wa.gov).

41. Morr, A. & Boers, N. Detection of approaching critical transitions in natural systems driven by red noise. *Phys. Rev. X* **14**, DOI: 10.1103/PhysRevX.14.021037 (2024).

42. Heßler, M. & Kamps, O. Do inner Greenland's melt rate dynamics approach coastal ones?, DOI: 10.48550/ARXIV.2411.07248 (2024). 2411.07248.

43. Veraart, A. J. *et al.* Recovery rates reflect distance to a tipping point in a living system. *Nature* **481**, 357–359, DOI: 10.1038/nature10723 (2011).

44. Grziwotz, F. *et al.* Anticipating the occurrence and type of critical transitions. *Sci. Adv.* **9**, DOI: 10.1126/sciadv.abq4558 (2023).

45. Bailer-Jones, C. A. L. 11.3 example of a numerical evidence calculation: is there evidence for a non-zero gradient? In *Practical Bayesian Inference. A Primer for Physical Scientists*, 225–254, DOI: 10.1017/9781108123891.012 (Cambridge University Press, 2017).

46. Seabold, S. & Perktold, J. Statsmodels: Econometric and statistical modeling with Python. In *9th Python in Science Conference* (2010).

47. Heßler, M. & Kamps, O. Anticipation of Oligocene's climate heartbeat by simplified eigenvalue estimation, DOI: 10.48550/ARXIV.2309.14179 (2023).

48. Wand, T., Heßler, M. & Kamps, O. Memory effects, multiple time scales and local stability in Langevin models of the S&P500 market correlation. *Entropy* **25**, 1257, DOI: 10.3390/e25091257 (2023).

49. Ehebrecht, F. *Anticipation of critical transitions in complex systems*. Master's thesis, Westphälische Wilhelms-Universität Münster (2017).

50. Hines, P., Cotilla-Sanchez, E. & Blumsack, S. Topological models and critical slowing down: Two approaches to power system blackout risk analysis. In *2011 44th Hawaii International Conference on System Sciences*, DOI: 10.1109/hicss.2011.444 (IEEE, 2011).

51. Cotilla-Sanchez, E., Hines, P. D. H. & Danforth, C. M. Predicting critical transitions from time series synchrophasor data. *IEEE Transactions on Smart Grid* **3**, 1832–1840, DOI: 10.1109/tsg.2012.2213848 (2012).

52. Ishii, M. & Ishii, H. DigitSeis: software to extract time series from analogue seismograms. *Prog. Earth Planet. Sci.* **9**, DOI: 10.1186/s40645-022-00508-0 (2022).

53. Corrado, R., Cherubini, A. M. & Pennetta, C. Early warning signals of desertification transitions in semiarid ecosystems. *Phys. Rev. E* **90**, DOI: 10.1103/physreve.90.062705 (2014).

54. Virtanen, P. *et al.* SciPy 1.0: fundamental algorithms for scientific computing in Python. *Nat. Methods* **17**, 261–272, DOI: 10.1038/s41592-019-0686-2 (2020).

55. Bronstein, I. N., Semendjajew, K. A., Musiol, G. & Mühlig, H. *Taschenbuch der Mathematik* (Harri Deutsch, Frankfurt a. M., 2008), 7 edn.
